# Elevated CD4^+^ T Cell Senescence Associates with Impaired Immune Responsiveness in Severe COVID-19

**DOI:** 10.14336/AD.2024.0214-2

**Published:** 2024-02-14

**Authors:** Jie Zhang, Chun Chang, Zhaoyuan Liang, Tingting Hu, Zhongnan Yin, Ying Liang, Ting Zhang, Yanling Ding, Xianlong Li, Xiaoyan Gai, Xiaoxue Yang, Xin Li, Xixuan Dong, Jiaqi Ren, Yafei Rao, Jun Wang, Jianling Yang, Lixiang Xue, Yongchang Sun

**Affiliations:** ^1^Department of Respiratory and Critical Care Medicine, Peking University Third Hospital, Beijing, China.; ^2^Center of Basic Medical Research, Institute of Medical Innovation and Research, Peking University Third Hospital, Beijing, China.; ^3^Biobank, Peking University Third Hospital, Beijing, China.

**Keywords:** CD4^+^ T cell senescence, COVID-19, spike-specific antibody, IL-2, CD40L, vaccine

## Abstract

Aging is a critical risk factor for unfavorable clinical outcomes among COVID-19 patients and may impact vaccine efficacy. However, whether the senescence of T cells is associated with severe COVID-19 outcome in elderly individuals is unclear. Using flow cytometry, we analyzed the frequency of senescent T cells (Tsens) in peripheral blood from 100 hospitalized elderly COVID-19 patients and compared differences between those with mild/moderate and severe/critical illness. We also assessed correlations between the percentage of Tsens and the quantity and quality of spike-specific antibodies by ELISA, neutralizing antibody test kit, and ELISPOT assay respectively, the cytokine production profile of COVID-19 reactive T cells, and plasma soluble factors by cytometric bead array (CBA). Our study found a significantly elevated level of CD4^+^ Tsens in patients with severe/critical disease compared to those with mild/moderate illness. Patients with a higher level of CD4^+^ Tsens (>19.78%) showed a decreased survival rate compared to those with a lower level (≤19.78%). This is more pronounced among patients with breakthrough infections. The percentage of CD4^+^ Tsens was negatively correlated with spike-specific antibody titers, neutralization ability, and COVID-19 reactive IL-2^+^CD4^+^ T cells. In addition, spike-specific antibody levels were positively correlated with IL-2 producing T cells and plasma IL-2 amount. Mechanistically, with defective CD40L, T cells from patients with CD4^+^ Tsens>19.78% were unable to support B cell proliferation and differentiation. Our data demonstrate that the percentage of CD4^+^ Tsens in peripheral blood may serve as a reliable biomarker for the prognosis of severe COVID-19 patients, especially in breakthrough infections. Therefore, restoring the immune response of CD4^+^ Tsens may be key to preventing severe illness and improving vaccine efficacy in older adults.

## INTRODUCTION

Aging is a major risk factor for increased coronavirus disease 2019 (COVID-19) severity, adverse clinical outcomes, and potentially reduced vaccine efficacy. Older adults have exhibited the greatest susceptibility to COVID-19, with higher rates of hospitalization, severe illness, and mortality [1, 2]. Poor antibody responses in adults over 60 years of age were observed from clinical trials of mRNA and recombinant spike protein vaccines [3, 4]. However, some older adults still derive benefits from COVID-19 vaccination [5], indicating variability in antiviral immune responses among older populations.

It’s not clear why older adults have impaired immune responses following infection or vaccination. Recent evidence suggests that T cell senescence may attenuate humoral and cellular immunity to COVID-19 infection or vaccination. COVID-19 patients have exhibited increased CD8^+^ T cells expressing CD57, a marker of T cell senescence [6]. Additionally, compared to younger adults, older individuals have lower vaccine-induced, spike-specific antibodies and weaker T cell responses, inversely correlated with senescent CD8^+^ T cell populations [7-10]. While numerous studies have characterized such alterations, comprehensive investigations in patients over 60 years of age remain lacking. Therefore, urgent efforts are needed to delineate the extent to which senescent T cells may influence severe disease progression and suboptimal vaccine responses in older adults.

To understand potential roles of senescent T cells in COVID-19 disease progression and vaccine responses, we investigated senescent T cell populations, spike-binding antibody and T cell responses, plasma soluble mediators, disease severity, and clinical outcomes among COVID-19 patients with a mean age of greater than 80 years. Our data indicate that CD4^+^ T cell senescence, characterized by defective interleukin 2 (IL-2) production and CD40 ligand (CD40L) expression, may impair spike-specific antibody responses quantitatively and qualitatively, thereby promoting progression to severe disease. These results suggest that CD4^+^ T cell senescence may be used as a biomarker to predict compromised antiviral immunity in older adults, with implications for next-generation vaccine approaches tailored to this vulnerable demographic.

## MATERIALS AND METHODS

### Study approval

Procedures involving human participant sampling and experimentation were reviewed and approved by the Peking University Third Hospital Institutional Review Board (license number IRB00006761-M2022865), in accordance with ethical standards for human subject’s research.

### Study cohort

The inpatient cohort comprised subjects admitted from December 23rd, 2022, to January 19th, 2023, at Peking University Third Hospital (Beijing, China). Patients were tested positive for severe acute respiratory syndrome coronavirus 2 (SARS-CoV-2) infection by nucleic acid detection and were consecutively enrolled. Patients were divided into mild/moderate or severe/critical groups according to standard clinical classifications for COVID-19. Mild cases exhibited modest symptoms without imaging evidence of pneumonia. Moderate cases had persistent fever >3 days and/or respiratory rates <30 breaths/min and resting pulse oximetry O_2_ saturation >93% with COVID-19 pneumonia verified by imaging. Severe cases had more than one of the following conditions: respiratory rate ≥30 breaths/min; O2 saturation ≤93% on room air; or arterial O_2_ partial pressure to fraction of inspired O2 ratio ≤300 mm Hg. Critically ill patients required mechanical ventilation for respiratory failure, were in shock or multiorgan failure requiring intensive care. Demographic details, medical history, and clinical data upon admission were extracted from electronic records for all participants. Patients discharged from hospital were followed up for 1 year by telephone interview with patients or their family members.

### Sample collection, processing and immunocyte isolation

Four mL ethylenediaminetetraacetic acid (EDTA) anti-coagulated peripheral venous blood was collected from SARS-CoV-2 infected patients at Peking University Third Hospital immediately following confirmation of positivity by nucleic acid testing. Samples were centrifuged at 2000×g for 10 minutes. The plasma was aliquoted and frozen at -80°C. Residual fractions were incubated in red blood cell lysis buffer (BioLegend). Leukocytes were retained for immunophenotyping, while peripheral blood mononuclear cells (PBMCs) were isolated using Ficoll (Sigma) density gradient centrifugation.

### Flow cytometry

Immune cell subset identification (Panel S1), T cell senescence markers (Panels S2, S4, S5), and T cell activation status (Panel S3) were examined by multicolor flow cytometry using corresponding protein markers.

For surface marker staining (Panels S1 and S4), 5×10^5^ leukocytes were incubated with indicated fluorescently conjugated antibodies for 20 minutes at room temperature protected from light. To evaluate senescence-associated β-galactosidase (SA-β-Gal) activity (Panel S2), 1×10^6^ leukocytes were processed using a commercial kit (Dojindo Molecular Technologies) per manufacturer's protocol, followed by immunolabeling with delineated surface markers. For intranuclear protein detection (Panel S5), 1×10^6^ cells were surface stained, then fixed and permeabilized using the True-Nuclear™ Transcription Buffer Set (BioLegend) prior to intranuclear target labeling for 60 minutes under dark room temperature conditions.

Both SARS-CoV-2 specific and non-specific cytokine production was quantified in CD4^+^ and CD8^+^ T cell subsets (Panel S3). Non-specific stimulation entailed culturing 1×10^6^ PBMCs with phorbol 12-myristate 13-acetate (PMA, BioLegend) and brefeldin A (BioLegend) for 5 hours. Specific stimulation employed incubation of 2×10^6^ PBMCs with a recombinant SARS-CoV-2 spike protein (2 μg/mL, MabTech) for 24 hours alongside brefeldin A addition during the final 5 hours. Surface and intracellular staining was conducted as above using commercial fixation/permeabilization buffers (BioLegend). For CD40L detection (Panel S6), 1×10^6^ leukocytes were stimulated using PMA/ionomycin for 5 hours prior to surface labeling.

Data acquisition was carried out on a CytoFLEX S flow cytometer (Beckman Coulter) and analyzed using Kaluza and FlowJo software. Antibodies utilized are listed in Supplementary Table 1, and gating schema is outlined in Supplementary Fig. 1.

### Evaluation of SARS-CoV-2 Spike-specific IgG and IgM

96-well microplates (Thermo Fisher Scientific) were coated with recombinant wild-type (WT) SARS-CoV-2 spike protein or WT and Omicron BA.7 receptor binding domain (RBD) proteins (Sino Biological) at 1 μg/mL diluted in coating buffer. Plates were incubated overnight at 4°C before blocking with enzyme-linked immunosorbent assay (ELISA) assay diluent (BioLegend) for 2 hours at room temperature. Serum samples were serially diluted in blocking buffer and added to the antigen-coated plates and incubated for 1 hour at room temperature. After washing, horseradish peroxidase (HRP) conjugated goat anti-human IgM (Sigma Aldrich) or IgG (Invitrogen) secondary antibodies were added and incubated for 45 minutes. Reactions were developed using 3,3′,5,5′-tetramethylbenzidine (TMB) chromogenic HRP substrate (BioLegend) and stopped by addition of ELISA stop solution (Solarbio). Optical densities were obtained at 450 nm and 630 nm and quantified using a Spark multimode microplate reader (Tecan). Endpoint dilution titers were calculated from the optical density vs. serum dilution curves using a 0.15 OD450-630nm cutoff in GraphPad Prism.

### Evaluation of SARS-CoV-2 RBD-specific neutralizing antibodies

A commercial SARS-CoV-2 RBD neutralizing antibody test kit (Vazyme Biotech) was used to quantify SARS-CoV-2 RBD-specific neutralizing antibody in patient samples. Serum samples were diluted 1:10 by adding 8 μL of serum to 72 μL of dilution buffer provided in the kit. Subsequent procedures were conducted per manufacturer’s instructions. Briefly, diluted serum samples were incubated at 37°C for 20 minutes in microplates pre-coated with angiotensin converting enzyme 2 (ACE2) receptor protein. Plates were washed, then 100 μL TMB chromogenic substrate was added and incubated for 15 minutes at 37°C. Reactions were terminated by addition of 50 μL stop solution before measuring absorbance at 450 nm using a Spark multimode microplate reader (Tecan). The percent inhibition of SARS-CoV-2 RBD binding to ACE2 was calculated using the formula: Inhibition (%) = (1 - Sample OD450nm/Negative Control OD450nm) × 100%.

### Evaluation of SARS-CoV-2 specific T cell responses by interferon-γ (IFN-γ)

Cellular immune responses were assayed with patient PBMCs using commercial IFN-γ pre-coated enzyme-linked immunoblot (ELISPOT) kits (MabTech) per manufacturer's instructions. Briefly, plates were blocked with RPMI 1640 medium (HyClone) containing 10% fetal bovine serum for 30 minutes at room temperature. PBMCs were plated at 3×10^5^ cells/well and stimulated with a pool of peptides specific to spike protein (2 μg/mL, MabTech). PMA served as a positive control, while medium alone was the negative control. Following 24-hour incubation at 37°C with 5% CO_2_, plates were washed and incubated with a biotinylated IFN-γ detection antibody for 2 hours at room temperature. Spot-forming cells were visualized and enumerated using an AID ELISPOT counter (AID Diagnostika) after addition of the 3-amino-9-ethylcarbazole (AEC) enzymatic substrate.

### Quantification of plasma soluble mediators

Levels of select proinflammatory cytokines, chemokines, and CD8^+^/NK cell-associated factors were quantified in patient plasma samples (n=53) using a multiplexed microbead-based immunoassay (LEGENDPlex^TM^, BioLegend) per manufacturer's guidelines. The following pre-formulated panels were utilized: Human ProInflammatory Chemokine Panel 1, Human Chemokine Panel 2, and Human CD8/NK Panel. Data acquisition was performed using a CytoFLEX S flow cytometer (Beckman Coulter) followed by proprietary data analysis using software from BioLegend.

### In vitro T cell-mediated B cell proliferation and differentiation

CD3^+^ T cells were isolated by positive selection using magnetic-activated cell sorting from COVID-19 patients. Sorted cells were stimulated with CD3 (2 μg/mL) and CD28 (1 μg/mL) monoclonal antibodies (BioLegend) for 24 hours and irradiated (30 Gy) prior to co-culture. B cells were negatively selected using anti-CD19 magnetic beads (Miltenyi Biotec) from a healthy donor and labeled with 2 μM carboxyfluorescein succinimidyl ester (CFSE; BioLegend) dye. Irradiated T cells (2×10^5^) were co-cultured with CFSE-labeled B cells (1×10^5^) for 8 days at 37°C in 96-well flat bottom plates in the presence or absence of an anti-CD40 antibody (10 μg/mL; BioLegend). Cell culture supernatants were collected on day 8 for IgM quantification by ELISA (Cloud Clone Corp). Proliferation was evaluated by CFSE dilution using flow cytometry. Differentiation into plasmablasts was assessed by quantifying the frequency of IgD^low^CD38^hi^ cells in the CD19^+^ B cell gate (Panel S7).

### Statistical analysis

Statistical analysis was conducted using GraphPad Prism version 9.0 and SPSS version 23.0 software packages. All reported p-values are two-tailed, with p<0.05 considered statistically significant. Specific tests included: Student's t-test for normally distributed data, Mann-Whitney U test for non-normally distributed data, Chi-square (χ2) or Fisher’s exact tests for categorical variables, Kaplan-Meier survival analysis with Gehan-Breslow-Wilcoxon testing, and Spearman rank correlation for nonparametric correlations. Thresholds delineating high versus low senescent T cell burdens were determined by log-rank optimization.

**Figure 1.**
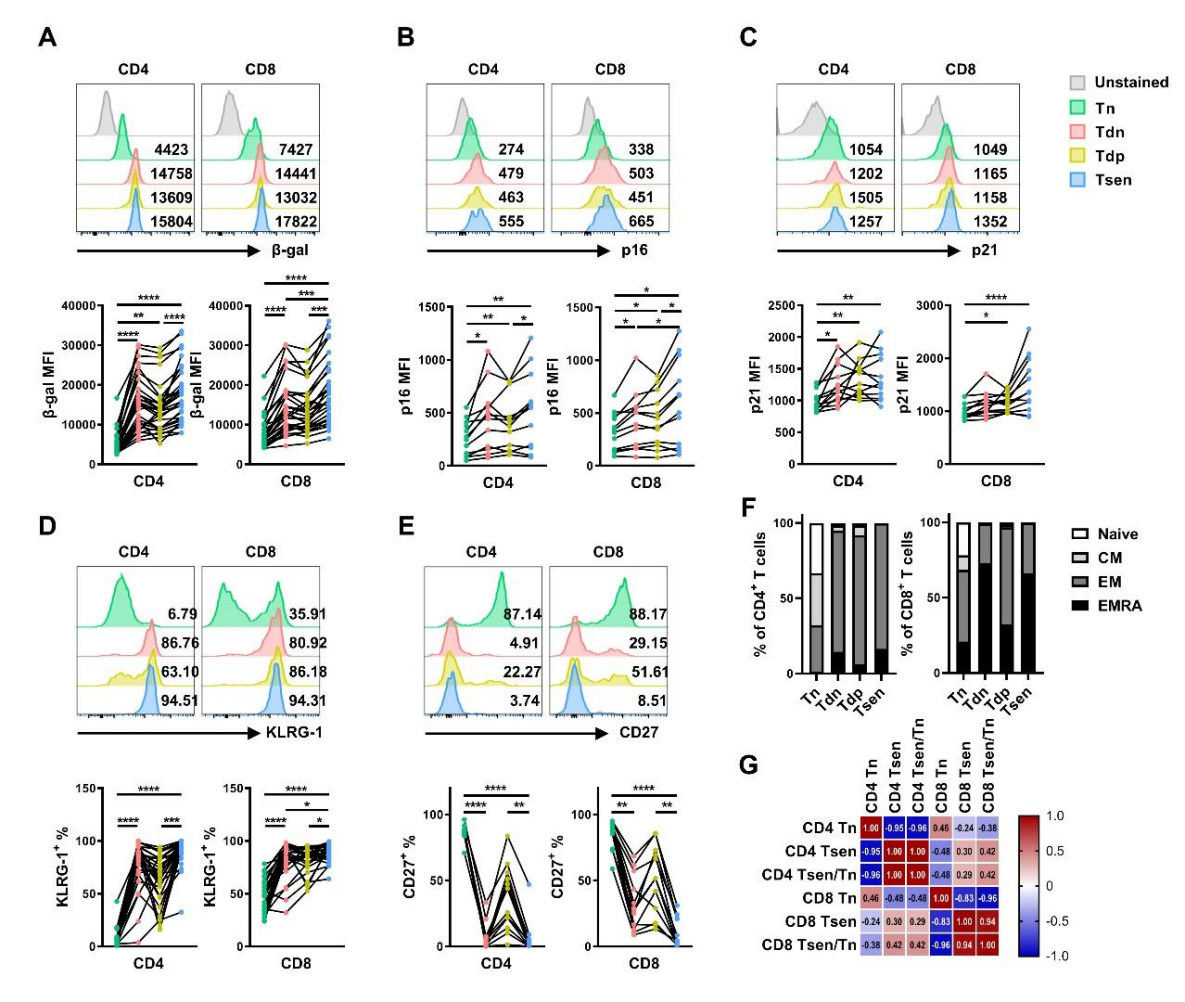
**Detailed phenotypic presentation of senescent T cells**. The representative flow cytometry plots and the statistic diagram show (**A**) The mean fluorescence intensity (MFI) of SA-β-gal (n=29); (**B**) the MFI of p16 (n=12); (**C**) the MFI of p21 (n=12); (**D**) the percentage of KLRG1^+^ T cells (n=29); (**E**) the percentage of CD27^+^ T cells (n=12) in the four subsets (Tn, Tdn, Tdp and Tsen) of CD4^+^ and CD8^+^ T cells. (**F**) The percentage of naive, central memory (CM), effect memory (EM), and effector memory cells re-expressing CD45RA (EMRA) T cells in the four subsets (Tn, Tdn, Tdp and Tsen) of CD4^+^ and CD8^+^ T cells (n=29). (**G**) Correlations between the percentage of 3 subsets (Tn, Tsen, Tsen/Tn) of CD4^+^ T cells and of CD8^+^ T cells (n=100). Groups were compared using paired t-test (**B**) or Mann-Whitney U-test (A, C, D, E). *, p< 0.05; **, p <0.01; ***, p <0.001; ****, p<0.0001.

## RESULTS

### Patient clinical characteristics

Demographics and clinical parameters of the study cohort are summarized in Table 1. The mean age was 80.10 ± 9.89 years, with 64% (64/100) being male. The mean body mass index (BMI) was 23.81 ± 3.91 kg/m2, with 38.5% (37/96) overweight (24.0 ≤ BMI ≤ 27.9 kg/m^2^) and 12.5% (12/96) obese (BMI ≥ 28.0 kg/m^2^) per Chinese BMI criteria. The most prevalent comorbidities included hypertension (52%), diabetes (25%), and cardiovascular diseases (24%). The most common symptoms were cough (85%), fever (82%), sputum production (80%), and dyspnea (60%). A greater proportion of severe/critically ill versus mild/moderate patients exhibited dyspnea upon admission (p=0.057). No other demographic or clinical features differed significantly between these groups.

**Table 1:** Demographics, Characteristics, and Clinical Features of Patients with Coronavirus Disease 2019^a^.

Characteristics	All cases(n=100)	Mild/Moderate (n= 36)	Severe/Critical (n=64)	*P*-value^b^
**Age, y(n)**	80.10±9.89	79.33±9.99	80.53±9.89	0.564
**Sex, male**	64(64%)	20(55.6%)	44(68.8%)	0.184
**BMI, kg/m^2^**	23.81±3.91(96)	23.32±3.82	24.07±3.96	0.379
**<18.5**	7(7.3%)	2(6.1%)	5(7.9%)	0.754
**18.5-23.9**	40(41.7%)	16(48.5)	24(38.1%)	
**24.0-27.9**	37(38.5%)	12(36.4)	25(39.7%)	
**≥28.0**	12(12.5%)	3(9.1%)	9(14.3%)	
**Smoking History, yes (n)**	35(35.0%)	10(27.8%)	25(39.1%)	0.256
**Any comorbidity**				
**Diabetes**	25(25%)	10(27.8%)	15(23.4%)	0.630
**Hypertension**	52(52.0%)	16(44.4%)	36(56.3%)	0.257
**Cardiovascular diseases**	24(24.0%)	6(16.7%)	18(28.1%)	0.198
**COPD**	11(11.0%)	2(5.6%)	9(14.1%)	0.331
**Asthma**	4(4.0%)	0(0%)	4(6.3%)	0.294
**aCCI**	4.8±1.23	4.92±1.05	4.85±1.33	0.825
**Signs and symptoms**				
**Fever**	82(82.0%)	29(82.9%)	53(84.1%)	0.871
**Cough**	85(85.0%)	30(83.3%)	55(85.9%)	0.726
**Sputum Production**	80(80.0%)	29(80.6%)	51(79.7%)	0.917
**Dyspnea**	60(60.0%)	17(47.2%)	43(67.2%)	0.057
**Medication**				
**Glucocorticoids**	84(84.0%)	26(72.2%)	58(90.6%)	0.016

BMI, body mass index; aCCI, age-adjusted Charlson Comorbidity Index; COPD, chronic obstructive pulmonary disease.

a Continuous variables were presented as mean ± SD (n); categorical variables are shown as n (%). Medication and respiratory support information was recorded during the entire hospital stay; other information was recorded at admission.

b P-values were from t-test for continuous data and from χ2 test for categorical data.

Laboratory tests findings are shown in Table 2. Severe/critical cases showed higher neutrophil counts (p=0.018) but lower lymphocyte counts (p=0.004) compared to mild/moderate cases. Disease severity was also associated with elevated D-dimer levels (p=0.035) and procalcitonin (p=0.046), indicative of augmented coagulation and inflammation. No significant differences existed across groups in other measured parameters.

Compared to mild/moderate cases, more patients with severe/critical COVID-19 received glucocorticoid treatment (84%) over their hospital course. Specific therapeutic details by severity stratum are listed in Table 1.

### Increased CD4^+^ T cell senescence in severe/critical COVID-19 patients.

Canonical markers of T cell senescence include the loss of CD28 and gain of CD57 [11]. Hence, we delineated four T cell subsets based on CD28/CD57 expression: CD28^+^CD57^-^ (Tn), CD28^-^CD57^-^ (Tdn), CD28^+^CD57^+^ (Tdp), and CD28^-^CD57^+^ (Tsen, gating strategy in Supplementary Fig. 1). Tsen displayed the highest SA-β-Gal activity alongside upregulated expression of cell cycle inhibitors p16 and p21 compared to Tn, Tdn, and Tdp subsets (Fig. 1A-C). Tsen exhibited the highest levels of terminal differentiation marker KLRG-1 (Fig. 1D), but costimulatory receptor CD27 is downregulated (Fig. 1E). Phenotypically, Tsen was predominantly effector memory (EM; CCR7^-^CD45RA^-^) and effector memory cells re-expressing CD45RA (EMRA; CCR7^-^CD45RA^+^) cells, while Tn constituted naïve (CCR7^+^CD45RA^+^) and central memory (CM; CCR7^+^CD45RA^-^) subsets (Fig. 1F). Moreover, the frequency of Tn inversely correlated with Tsen percentages and Tsen/Tn ratios within both CD4^+^ and CD8^+^ compartments (Fig. 1G). We observed significant correlation between CD4^+^ and CD8^+^ Tsen levels (r=0.30, p=0.002; Fig. 1G), indicating coordinated senescence programming.

**Figure 2.**
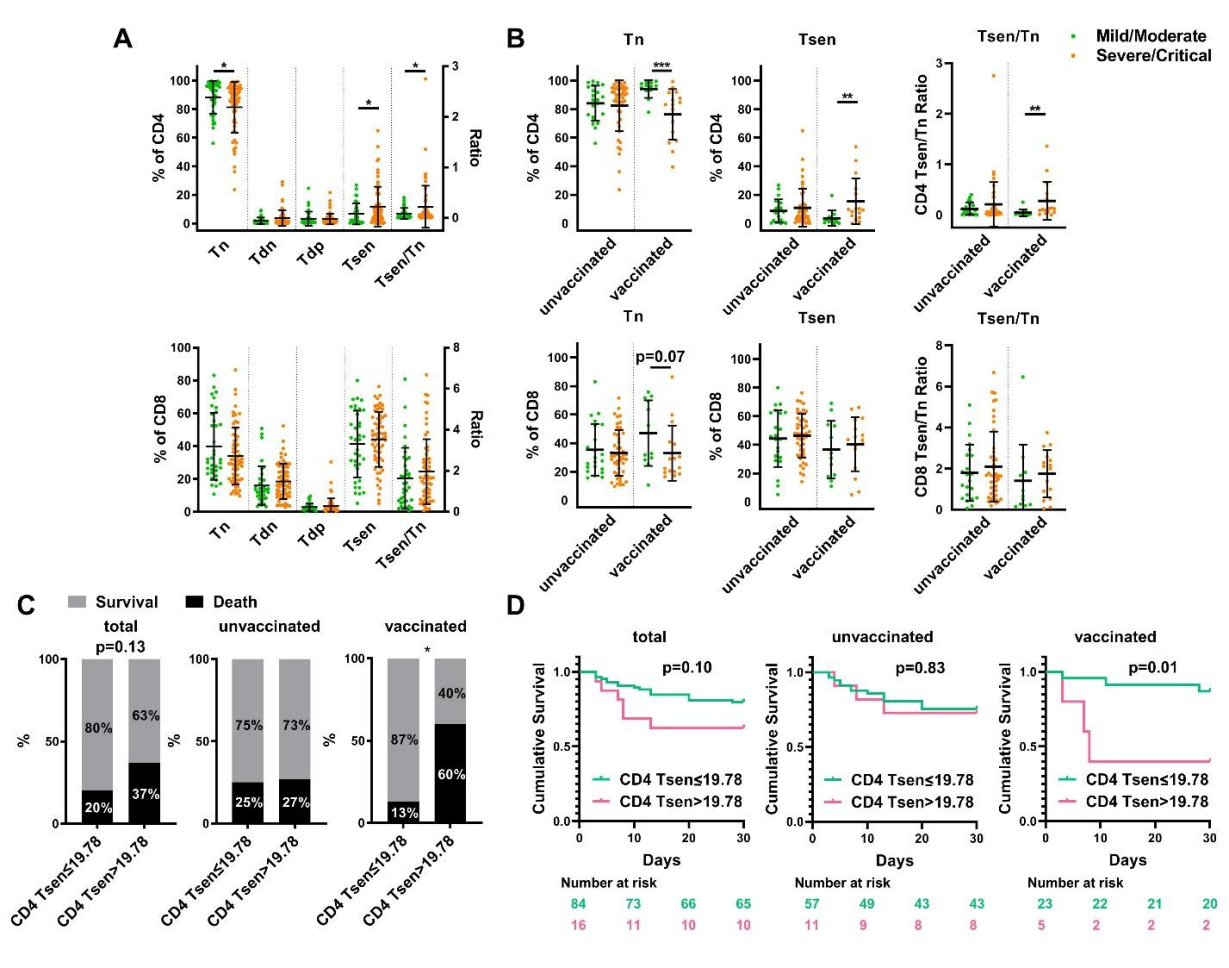
**Increased CD4^+^ Tsens in severe/critical COVID-19**. (**A**) The percentage of CD4^+^ and CD8^+^ T cell subsets: CD28^+^CD57^-^ (Tn), CD28^-^CD57^-^(Tdn), CD28^+^CD57^+^ (Tdp), and CD28^-^CD57^+^ (Tsen) and Tsen/Tn ratio in mild/moderate patients (n=36) compared to severe/critical patients (n=64). (**B**) The percentage of CD4^+^ and CD8^+^ Tn, Tsen and Tsen/Tn ration in unvaccinated group (mild/moderate: n=22 versus sever/critical: n=46) and vaccinated group (mild/moderate: n=12 versus sever/critical: n=16). Groups were compared using Mann-Whitney U-test. Bars show mean with SD. (**C**) Survival rates in patients with COVID-19 stratified by levels of CD4 Tsen (the entire cohort: CD4 Tsen≤19.78%, n=84 versus CD4 Tsen>19.78%, n=16; unvaccinated group: CD4 Tsen≤19.78%, n=57 versus CD4 Tsen>19.78%, n=11; vaccinated group: CD4 Tsen≤19.78%, n=23 versus CD4 Tsen>19.78%, n=5). P values for difference between survival rates were calculated using Fisher exact test. (**D**) Kaplan-Meier 30-day survival curves for COVID-19 patients with CD4 Tsen >19.78% versus CD4 Tsen ≤19.78% (the entire cohort: CD4 Tsen≤19.78%, n=84 versus CD4 Tsen>19.78%, n=16; unvaccinated group: CD4 Tsen≤19.78%, n=57 versus CD4 Tsen>19.78%, n=11; vaccinated group: CD4 Tsen≤19.78%, n=23 versus CD4 Tsen>19.78%, n=5). P values for difference between two groups of curves were calculated by the log rank test. *, p< 0.05; **, p <0.01; ***, p <0.001.

To interrogate relationships between T cell senescence and COVID-19 severity, we divided patients into mild/moderate (n=69) or severe/critical (n=31) illness groups. While CD8^+^ T cell subset distributions were comparable across groups, the severe/critical group showed reduced CD4^+^ Tn frequencies coupled with elevated CD4^+^ Tsen percentages, yielding higher Tsen/Tn ratios (CD4^+^ Tn, p=0.0303; CD4^+^ Tsen, p=0.0401; CD4^+^ Tsen/Tn, p=0.0334; Fig. 2A). We further investigated unvaccinated (n=68) and vaccinated (n=28) patients to access relationships between senescence and breakthrough infections after immunization. The association between CD4^+^ T cell senescence and severity was pronounced to vaccinated patients, wherein decreased Tn and elevated Tsen levels associated with critical illness post-vaccination (CD4^+^ Tn, p=0.0007; CD4^+^ Tsen, p=0.0044; CD4^+^ Tsen/Tn, p=0.0037; Fig. 2B). This phenomenon is not observed in unvaccinated patients (Fig. 2B).

CD4^+^ Tsen levels correlated positively with age, obesity, and age-related comorbidities like cardiovascular diseases and chronic obstructive pulmonary diseases. However, these associations plateaued in the very old (>80 years) cohort, indicating senescence may reach a maximal threshold (Supplementary Fig. 2A). Elevated CD8^+^ Tsen is associated with systemic inflammation (C-reactive protein) and tissue damage (lactate dehydrogenase) but declined platelet counts (Supplementary Fig. 2A). Among circulating leukocytes, CD8^+^ and CD4^+^ Tsen inversely correlated with lymphocyte levels and antigen-presenting cells (including monocytes and dendritic cells) which may enable disease progression (Supplementary Fig. 2B-E).

**Figure 3.**
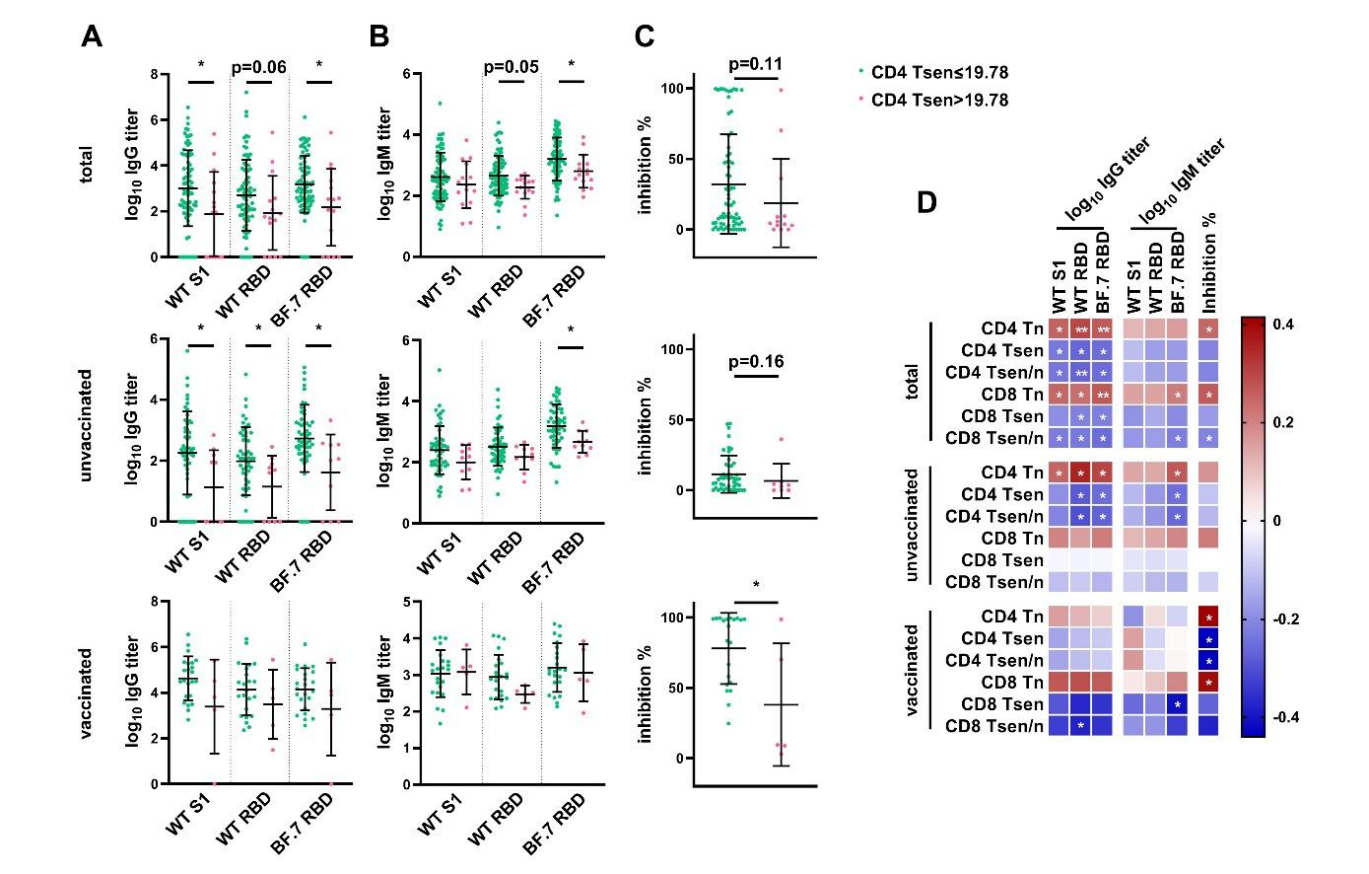
**Higher levels of senescent CD4^+^ T cells is correlated with lower spike-specific antibody levels**. Plasma IgG (**A**) and IgM (**B**) against the S1 domain of original SARS-CoV-2 strain (WT S1), the RBD domain of original strain (WT RBD) and the Omicron variants BF.7 (BF.7 RBD), and neutralization antibody inhibition rate (**C**) in COVID-19 patients with different level of CD4 Tsen (the entire cohort: CD4 Tsen≤19.78%, n=80 versus CD4 Tsen>19.78%, n=15; unvaccinated group: CD4 Tsen≤19.78%, n=53 versus CD4 Tsen>19.78%, n=10; vaccinated group: CD4 Tsen≤19.78%, n=23 versus CD4 Tsen>19.78%, n=5). (**D**) Correlations between the percentage of senescent T cells and IgG and IgM titers (against WT S1, WT RBD and BF.7 RBD), inhibition rate (the entire cohort: n=95, unvaccinated group: n=63; vaccinated group: n=28). Statistical comparisons across cohorts were performed using the Mann-Whitney test. Spearman’s rank correlation was used to identify relationships between two variables. R values are indicated by color. Significant correlations were indicated by white asterisks. *, p< 0.05; **, p <0.01.

Subsequently, we examined associations between CD4^+^ Tsen and survival. The median CD4^+^ Tsen level was 5.19% (range 0.01-64.95%). By a data-driven cutoff (>19.78%), 16% of the patients were classified as having high CD4^+^ Tsen. These individuals showed a higher rate of severe/critical illness (87% vs. 60%, p=0.045) despite comparable clinical features (Supplementary Tables 2-3). High CD4^+^ Tsen was associated with reduced survival at hospital (80% vs. 63%, p=0.13), particularly among the vaccinated patients (87% vs. 40%, p=0.02) but not among the unvaccinated (Fig. 2C-D). However, no association was observed between levels of CD4^+^ Tsen and long-term (1 year) survival (Supplementary Fig. 4).

Collectively, these results indicate that CD4^+^ T cell senescence may serve as a prognostic biomarker for severe COVID-19 and diminished vaccine responses, especially among vulnerable elderly populations.

**Table 2:** Laboratory Characteristics on Admission for Severely and Critically Ill Patients with Coronavirus Disease 2019^a^.

Characteristics	All cases(n=100)	Mild/Moderate (n=36)	Severe/Critical (n=64)	*P*-value^b^
**Blood routine**	
**White blood cell count, 10^9^/L**	7.56±2.9	7.13±2.25	7.79±3.21	0.277
**<3.5**	2(2.0%)	1(2.8%)	1(1.6%)	0.501
**3.5~9.5**	75(75.0%)	29(80.6%)	46(71.9%)	
**>9.5**	23(23.0%)	6(16.7%)	17(26.6%)	
**Neutrophil count, 10^9^/L**	6.39±2.77	5.61±1.93	6.82±3.07	0.018
**Lymphocyte count, 10^9^/L**	0.78±0.48	0.99±0.61	0.66±0.34	0.004
**Platelet count, 10^9^/L**	214.62±81.04	234.44±75.68	203.47±82.39	0.066
**Hemoglobin, g/L**	121±28.41	122.08±18.92	120.39±32.68	0.776
**Inflammatory markers**				
**Procalcitonin, ng/mL**	0.38±1.10	0.14±0.18	0.51±1.35	0.046
**<0.1**	53(54.1%)	23(63.9%)	30(48.4%)	0.324
**01~0.3**	30(30.6%)	9(25.0%)	21(33.9%)	
**>0.3**	15(15.3%)	4(11.1%)	11(17.7%)	
**C-reactive protein, mg/L**	24.76±40.66	24.77±37.11	24.75±42.77	0.998
**≤8**	41(42.7%)	17(50.0%)	24(38.7%)	0.285
**>8**	55(57.3%)	17(50.0%)	38(61.3%)	
**Coagulation function**				
**D-dimer, ug/mL**	2.77±5.00	1.53±2.85	3.41±5.73	0.035
**≤age/100**	47(49.0%)	21(63.6%)	26(41.3%)	0.037
**> age/100**	49(51.0%)	12(36.4%)	37(58.7%)	
**Serum biochemical indicators**				
**Serum albumin level, g/L**	31.89±4.87	33.04±4.11	31.24±5.17	0.075
**Creatinine, μmol/L**	98.34±92.85	96.75±71.64	99.23±103.42	0.899
**Serum urea nitrogen, mmol/L**	9.96±8.53	9.71±7.00	10.10±9.34	0.828
**Total bilirubin, μmol/L**	12.02±5.66	11.31±3.81	12.41±6.47	0.352
**Alanine Aminotransferase, U/L**	37.49±38.22	33.48±39.71	39.74±37.50	0.435
**Aspartate Aminotransferase, U/L**	42.47±35.11	38.26±28.96	44.84±38.15	0.371
**Creatine kinase, U/L**	109.06±182.15	88.56±70.43	121.16±223.13	0.397
**Creatine kinase-MB, U/L**	15.56±31.62	18.06±33.74	14.13±30.53	0.554

a Continuous variables were presented as median (interquartile range); categorical variables are shown as n (%).

b P-values were from t-test for normally distributed continuous data and from Mann-Whitney U test for abnormally distributed continuous data. P-values were from χ2 test for categorical data.

### Elevated CD4^+^ T cell senescence was associated with diminished COVID-19 specific humoral immunity.

Virus-neutralizing antibodies have been implied to offer protection against infection [12, 13]. To interrogate relationships between T cell senescence and virus-specific antibody production, we quantified IgG and IgM titers against WT SARS-CoV-2 spike protein (S1) and RBD, alongside the Omicron BA.7 variant RBD, in patient plasma. Since the contemporary epidemic strain was BA.7, anti-BA.7 RBD titers exceeded those against WT S1 and RBD (Supplementary Fig. 5A). Vaccinated patients exhibited higher WT spike-specific IgG and IgM levels, though anti-BA.7 RBD IgM was comparable (Supplementary Fig. 5B-C), indicating limited Omicron variant protection by vaccination in this older cohort.

Patients with high CD4^+^ Tsen (>19.78%) displayed an approximately 90% reduction in median anti-WT S1 IgG titers compared to that of the low CD4^+^ Tsen group (medians of 89 versus 870; Fig. 3A). Moreover, median anti-BA.7 RBD IgG and IgM levels were profoundly diminished among those with elevated CD4^+^ T cell senescence (Fig. 3A-B). This phenomenon was observed across both primary infection and breakthrough subgroups (Fig. 3A-B).

Subsequently, we evaluated the functional capacity of serum antibodies to neutralize ancestral SARS-CoV-2 variants. Neutralization potency was enhanced in vaccinated patients comparing to that of the unvaccinated (70.28%±32.74% versus 10.68%±13.08% inhibition, p<0.0001; Supplementary Fig. 5D). However, within the vaccinated cohort, individuals exhibiting high CD4^+^ Tsen displayed marked impairment in viral neutralization activities comparing to the low CD4^+^ Tsen group (37.99%±43.59% versus 77.97%±25.25%, p=0.049; Fig. 3C). Similar differences were observed among the total population and unvaccinated patients (Fig. 3C).

Anti-spike IgG, RBD IgG, and BA.7 RBD IgG levels inversely correlated with frequencies of both CD4^+^ and CD8^+^ Tsen cells (Fig. 3D). Notably, anti-BA.7 RBD IgG and IgM levels are associated with CD4^+^ Tsen among unvaccinated individuals. Viral neutralization capacity correlated positively with CD4^+^ Tn but negatively with CD4^+^ Tsen, particularly in the vaccinated subgroup (Fig. 3D).

**Figure 4.**
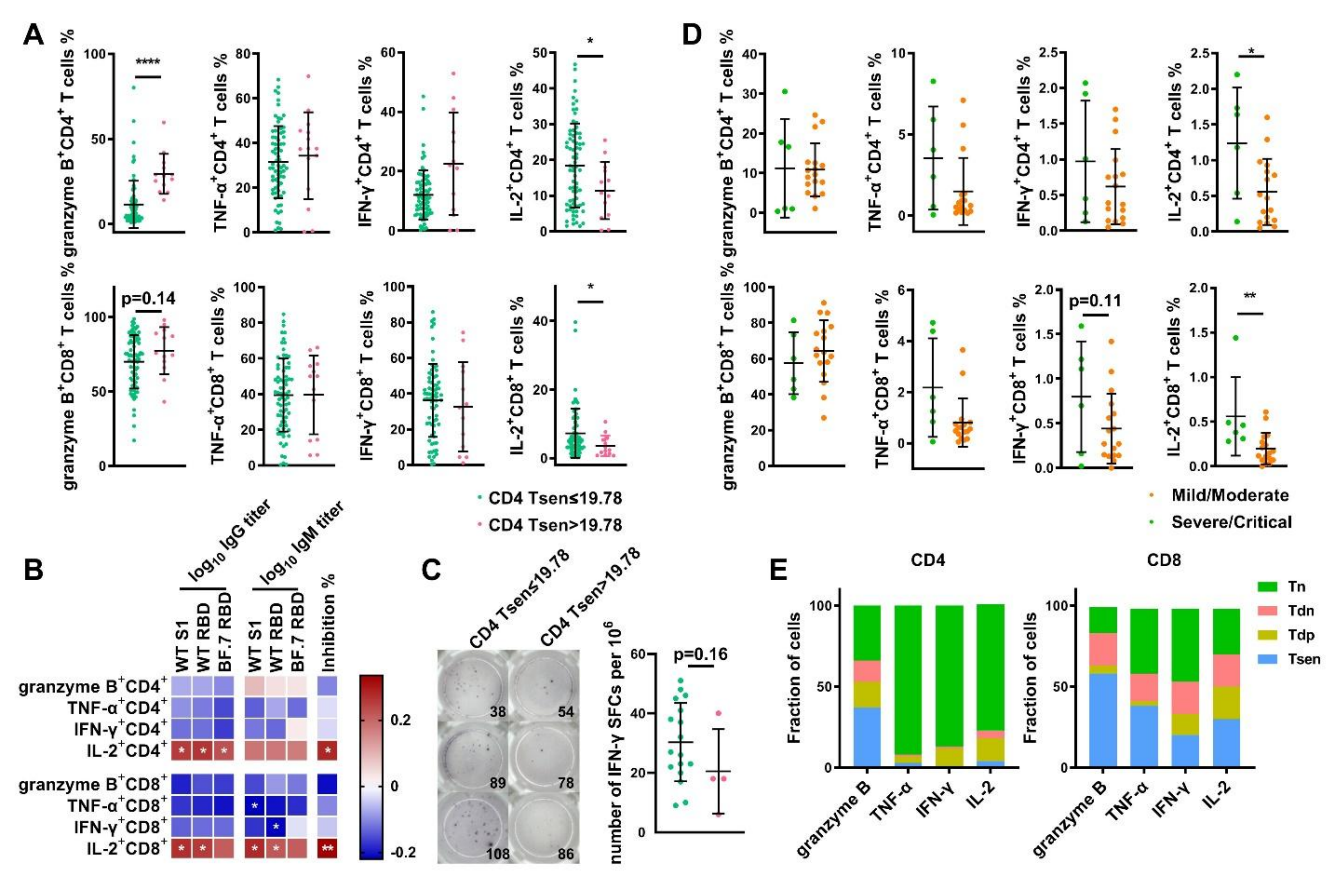
**Higher granzyme B^+^ and lower IL-2^+^ T cells are associated with defects in antibody levels**. (**A**) Frequency of cytokine^+^ T cells (after PMA stimulation) in patients with CD4 Tsen ≤19.78% group (n=74) and CD4 Tsen >19.78% group (n=13). (**B**) Correlations between the percentage of cytokine^+^ T cells and plasma IgG and IgM against the S1 domain of original SARS-CoV-2 strain (WT S1), the RBD domain of original strain (WT RBD) and the Omicron variants BF.7 (BF.7 RBD, n=83), inhibition rate (n=74). (**C**) Frequency of IFN-γ spot-forming cells (SFC) after stimulation with Spike in patients with CD4 Tsen ≤19.78% group (n=17) and CD4 Tsen >19.78% group (n=4). The number represents the patient number. (**D**) Frequency of cytokine^+^ T cells (after Spike stimulation) in patients with mild/moderate illness (n=6) and severe/critical illness (n=17). (**E**) The frequency of Tn, Tdn, Tdp and Tsen (defined using the markers CD57 and CD28) within spike-specific cytokine^+^ T cells (n = 21). Groups were compared using Mann-Whitney U-test. Bars show mean with SD. Spearman’s rank correlation was used to identify relationships between two variables. R values are indicated by color. Significant correlations were indicated by white asterisks. *, p< 0.05; **, p <0.01; ****, p <0.0001.

In summary, the accumulation of senescent CD4^+^ T cells may hamper spike-specific antibody production and its neutralizing activity, leading to more severe disease and mortality risk among older COVID-19 patients.

### Higher granzyme B and lower IL-2 were associated with impaired humoral immunity.

To further characterize the functional phenotype of CD4^+^ Tsen cells, we evaluated the cytokine production profiles between patients with high or low CD4^+^ Tsen frequencies. The CD4^+^ Tsen high group displayed reduced IL-2^+^CD4^+^ T cells but elevated granzyme B^+^CD4^+^ T cells relative to their CD4^+^ Tsen low counterparts (Fig. 4A). Consistent with these observations, severe/critical COVID-19 patients exhibited expanded granzyme B^+^ T cell populations but fewer IL-2^+^ T cells comparing to that of mild/moderate cases (Supplementary Fig. 6A). Both CD4^+^ and CD8^+^ Tsen levels positively correlated with granzyme B^+^ T cell frequencies and negatively correlated with IL-2^+^ T cell levels (Supplementary Fig. 6B). Relative to Tn, Tdn, and Tdp subsets, Tsen preferentially produced granzyme B, tumor necrosis factor α (TNF-α), and IFN-γ, while IL-2 expression was compromised (Supplementary Fig. 6C). Notably, the frequency of IL-2^+^ T cells was associated positively with spike-binding antibody production and neutralization capacity (Fig. 4B), implicating defective IL-2 expression by CD4^+^ Tsen as a potential contributor to impaired humoral immunity.

Subsequently, we measured SARS-CoV-2 specific T cell responses using IFN-γ ELISPOT assays after stimulating PBMCs with spike peptides. The overall spike-specific T cell response was lower among those with high levels of CD4^+^ Tsen compared to groups with low CD4^+^ Tsen (18 versus 27 spots, p=0.16; Fig. 4C). Furthermore, we identified T cell subsets utilizing intracellular cytokine staining following spike peptide stimulation. While group sizes precluded comparing CD4^+^ Tsen high (n=2) versus low (n=21) cohorts, we instead stratified the patients by mild/moderate (n=14) versus severe/critical (n=6) illness. In agreement with the ELISPOT data, severe/critical patients showed reduced levels of spike-specific IFN-γ^+^, and TNF-α^+^ CD4^+^ and CD8^+^ T cells (Fig. 4D). Similarly, the frequency of IL-2^+^ spike-specific CD4^+^ and CD8^+^ T cells was higher in the mild/moderate group (Fig. 4D). No differences in disease severity were observed in granzyme B production (Fig. 4D). Further analysis revealed that the majority of IL-2^+^, TNF-α^+^, and IFN-γ^+^ spike-reactive cells are Tn, while granzyme B^+^ cells as predominantly Tsen (Fig. 4E).

Collectively, these data indicate that upregulated granzyme B and downregulated IL-2 are features of CD4^+^ T cell senescence that may be associated with impaired humoral immunity against SARS-CoV-2.

**Figure 5.**
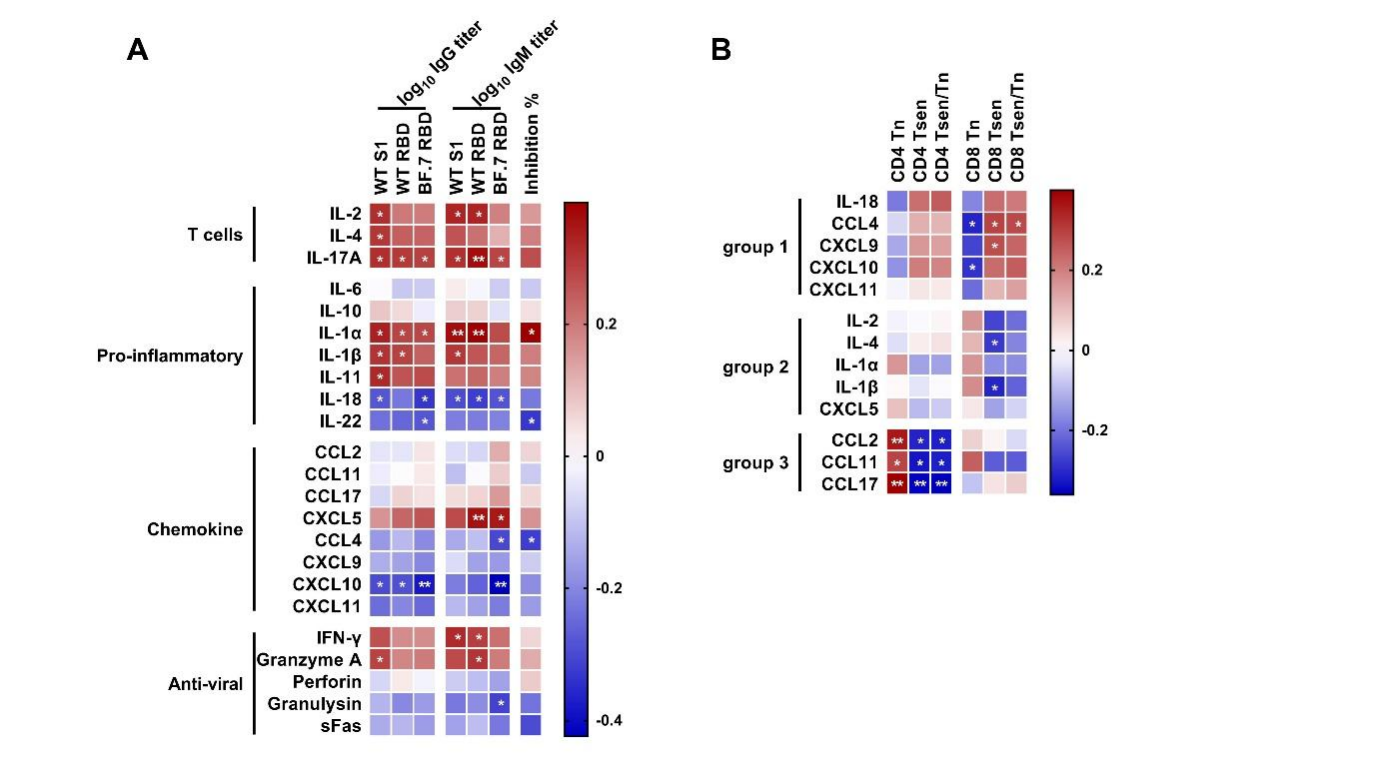
**The correlation between plasma levels of soluble factors and spike-specific antibody levels, neutralization ability as well as the percentage of senescent T cells**. (**A**) Correlation analysis between IgG and IgM titers (against WT S1, WT RBD and BF.7 RBD), inhibition rate and plasma levels of soluble factors (IL-2, IL-4, IL-17A, IL-6, IL-10, IL-1α, IL-1β, IL-11, IL-18, IL-22, CCL2, CCL11, CCL17, CXCL5, CCL4, CXCL9, CXCL10, CXCL11, IFN-γ, granzyme A, perforin, granulysin, sFas). The function mediated by each plasmatic molecule was indicated on the left. (**B**) Correlations between the percentage of senescent T cells and plasma levels of soluble factors (group 1: IL-18, CCL4, CXCL9, CXCL10, CXCL11; group 2: IL-2, IL-4, IL-1α, IL-1β, CXCL5; group 3: CCL2, CCL11, CCL17). Data were collected from 53 COVID-19 infected patients, except the inhibition rate was detected in 42 patients. Spearman’s rank correlation was used to identify relationships between two variables. R values are indicated by color. Significant correlations were indicated by white asterisks. *, p<0.05; **, p<0.01.

### Associations between T cell senescence, humoral immunity, and systemic inflammation.

We measured inflammatory mediator profiles using a 39-plex cytokine array from plasma of 53 COVID-19 patients. Higher IL-6 levels were observed in severe/critical cases compared to that in mild/moderate cases (71.54±23.36 pg/mL vs 22.02±9.565 pg/mL, p=0.130; Supplementary Table 4). As IL-6 represents a canonical senescence-associated secretory phenotype (SASP) factor, this result highlights potential involvement of inflammatory pathways in disease progression.

Next, we examined relationships between spike-specific antibody levels and circulating cytokines/ chemokines grouped by primary bioactivity (Fig. 5A). As anticipated, IL-2 levels correlated positively with spike-specific antibody levels. Other T cell-related cytokines (IL-4, IL-17A) that stimulate B cell responses and antibody production [14-16] showed similar associations. Notably, pro-inflammatory cytokines IL-1α/β and IL-11 positively correlated with spike-specific IgG/IgM, while IL-18 and IL-22 showed the reverse correlations. Regarding chemokines, the spike-specific IgG/IgM were associated positively with C-X-C motif ligand (CXCL) 5 but negatively with C-C motif ligand (CCL) 4, CXCL9, CXCL10, and CXCL11, which were reportedly upregulated in severe COVID-19 [17]. Complex correlations of spike-specific antibodies with antiviral effectors were also observed, positively with IFN-γ and granzyme A, but negatively with perforin and granulysin.

**Figure 6.**
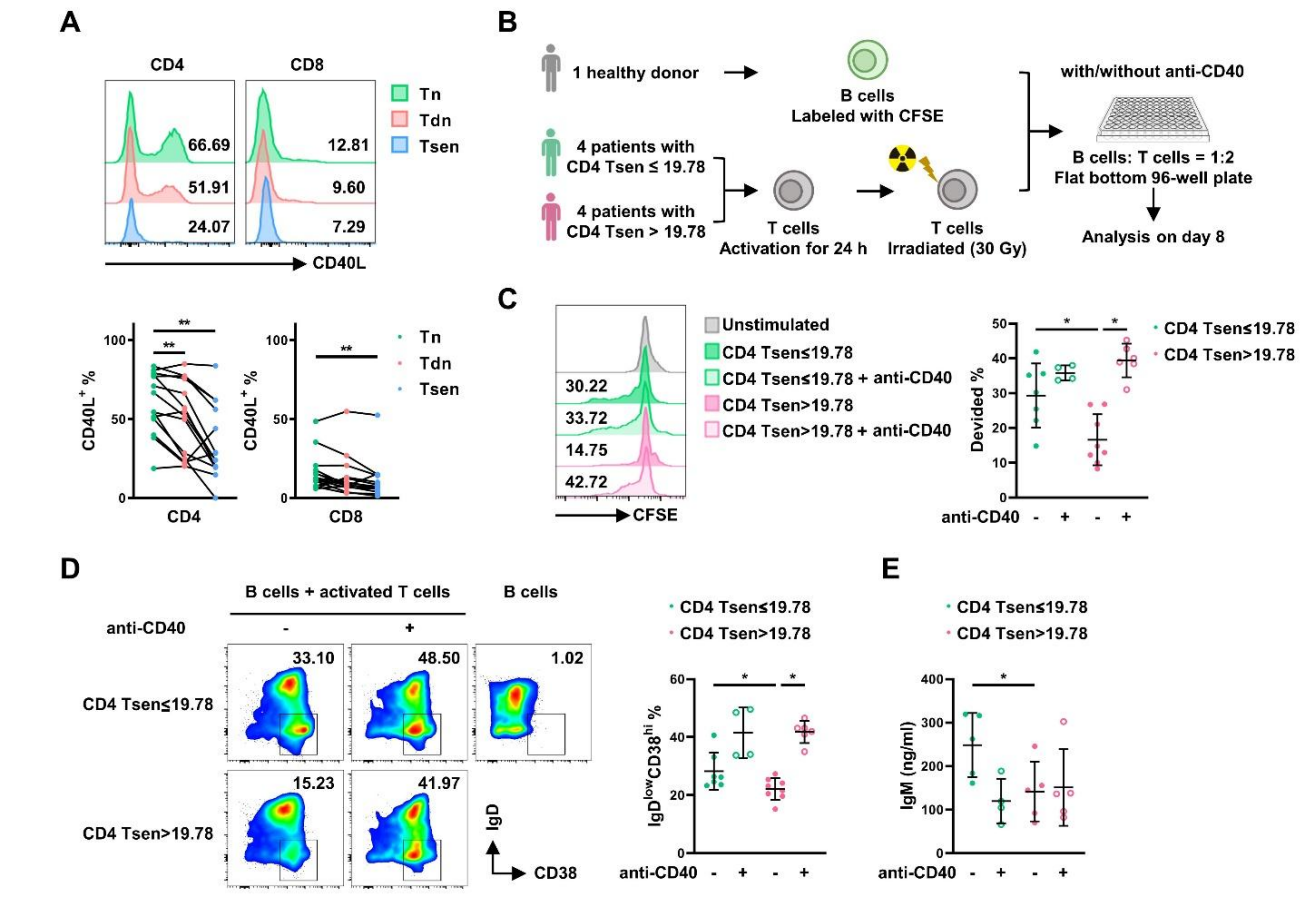
**CD4^+^ Tsens were defect in T cell-mediated B cell proliferation and differentiation**. (**A**) The representative flow cytometry plots and the statistic diagram show the percentage of CD40L^+^ T cells (n=15) in the three subsets (Tn, Tdn, and Tsen) of CD4^+^ and CD8^+^ T cells on stimulation with PMA/ionomycin for 5 h. (**B**) The CD3^+^ T cells from 4 CD4^+^ Tsen≤19.78% and 4 CD4^+^ Tsens>19.78% COVID-19 patients were stimulated with CD3 mAb (2 μg/mL) and CD28 mAb (1 μg/mL) for 24 h and irradiated (30 Gy). T cells were then cocultured with CFSE-labeled B cells with or without anti-CD40 for 7 days. (**C**) The proliferation of B cells was assessed based on CFSE dilution. (**D**) A representative density plot of the expression of CD38 and IgD on CD19^+^ cells and the statistic graphic were shown. (**E**) the supernatant from the same cocultures was analyzed by ELISA for secretion of IgM. 2 repeats from 4 patients for each group. Groups were compared using paired Mann-Whitney U-test. Bars show mean with SD.*, P < 0.05; **, P < 0.01; ***, P < 0.001; ****, P < 0.0001.

Subsequently, we examined associations between inflammatory mediators and T cell senescence. Correlations emerged among three groups (Fig. 5B). Group 1 (IL-18, CCL4, CXCL9, CXCL10, and CXCL11) had inverse correlation with antibody responses, but positive CD4^+^/CD8^+^ Tsen correlation. Group 2 (IL-2, IL-4, IL-1α/β, and CXCL5) positively correlated with antibodies but negatively correlated with senescent T cell frequencies. Lastly, group 3 (CCL2, CCL11, CCL17) exhibited positive CD4^+^ Tsen correlations without antibody associations. Collectively, these results imply that group 1 and 2 molecules may contribute to senescence-associated humoral immune defects, while group 3 molecules could act through separate pathways.

### Impaired B cell helper function of senescent CD4^+^ T cells

CD40L expression on activated T cells engages CD40 on B cells, triggering proliferation, differentiation, and antibody production during cognate interactions [18, 19]. We thus examined CD40L profiles across T cell subsets and found that the highest frequencies on Tn cells following PMA/ionomycin stimulation. In contrast, Tsen cells showed the lowest CD40L expression (Fig. 6A). We therefore hypothesized that senescent T cells may be intrinsically defective at supporting B cell responses due to constrained CD40L expression.

To test this hypothesis, we isolated T cells from patients grouped into high (n=4) or low (n=4) CD4^+^ Tsen frequencies. Isolated T cells were activated *in vitro* and co-cultured with CFSE-labeled B cells from a healthy donor (Fig. 6B). On day 8, B cell proliferation was quantified via CFSE dilution and differentiation by IgD^low^CD38^hi^ staining on CD19^+^ cells alongside secreted IgM. In the absence of T cells, B cells remained quiescent. T cells from high CD4^+^ Tsen group showed reduced capacity to induce B cell proliferation compared to low CD4^+^ Tsen group (Fig. 6C). The frequency of IgD^low^CD38^hi^CD19^+^ plasmablasts was also diminished in high CD4^+^ Tsen group (Fig. 6D), yielding lower IgM level (Fig. 6E). Addition of exogenous anti-CD40 antibody partially rescued defects in proliferation and differentiation conferred by senescent T cells (Fig. 6C-E).

Taken together, our data demonstrate that CD4^+^ T cell senescence links to impaired CD40L expression and defective capacity to provide B cell helper signals.

## DISCUSSION

In this study, we investigated the role of T cell senescence in elderly patients with primary and breakthrough COVID-19 infections. We demonstrated that elderly patients with severe/critical illness exhibited expanded CD4^+^ Tsen compared to those with mild/moderate disease. Moreover, we found a higher in-hospital mortality rate among those with CD4^+^ Tsen frequencies above 19.78%, and this was more pronounced in vaccinated individuals. Frequencies of CD4^+^ Tsen correlated inversely with SARS-CoV-2 spike-specific IgG/IgM titers and neutralization activity. Furthermore, frequencies of IL-2^+^ T cells and plasma IL-2 levels were positively associated with antibody levels. Mechanistically, CD4^+^ Tsen cells displayed constrained CD40L expression resulting in defective capacity to support B cell proliferation and differentiation. Our data demonstrated that circulating CD4^+^ Tsen levels may serve as a reliable biomarker to predict COVID-19 severity and prognosis in elderly patients, especially in breakthrough infections.

Immune responses to SARS-CoV-2 infection or vaccination decline with advanced age, cytomegalovirus exposure, and age-associated comorbidities [7, 20, 21]. However, these factors showed no relationship with disease severity or breakthrough infection rate in our elderly cohort (Table 1), indicating a lack of differentiation among these aged demographics. Interestingly, we identified preferential accrual of highly differentiated CD4^+^ but not CD8^+^ senescent T cells in severe/critical relative to mild/moderate patients (Fig. 2A-B) alongside increased mortality above a CD4^+^ senescence threshold (>19.78%, Fig. 2C). In agreement with prior evidence linking short leukocyte telomeres to poor COVID-19 outcomes [1], our data implicate CD4^+^ T cell aging as a predominant driver for immunopathology in patients with advanced ages where additional age-related immune remodeling may have been plateaued.

CD8^+^ T cell senescence outpaces CD4^+^ counterparts over time [22, 23]. While SARS-CoV-2-reactive CD8^+^ T cell memory compartments show particular vulnerabilities to aging [20], our findings highlight CD4^+^ T cell integrity as central to antiviral responses in this elderly cohort. Emerging evidence indicates that rapid and robust immune reactions following infection may mitigate severe progression [24]. Aligning with the critical role for T cell immunity in long-term vaccine protection [25-27], our results suggest that COVID-19 vaccines may reduce serious breakthrough infections in seniors with lower CD4^+^ T cell senescence. For those exceeding the prognostic CD4^+^ aging threshold, early antiviral or neutralizing antibody treatment may provide effective treatment.

There are several factors underlying the heightened susceptibility to severe COVID-19 among elderly individuals with accumulated senescent T cell burdens. On the one hand, the constrained clonal diversity accompanying advanced age [28] together with defects in T cell receptor signaling intrinsic to senescent cells [29] likely erode spike-specific T cell immunity. However, while highly differentiated CD4^+^ subsets display enhanced capacity for cytotoxic mediator elaboration like granzyme B, our findings raise the possibility that this represents a double-edged sword - facilitating viral clearance at the cost of bystander pulmonary damage [30]. On the other hand, CD4^+^ T cell senescence additionally drives impaired humoral support. Beyond restrained vaccine-induced antibody production [7, 9], we demonstrate a strong association between CD4^+^ but not CD8^+^ senescence markers and circulating SARS-CoV-2 spike-binding antibody titers and neutralization potential in these infected seniors (Fig. 3). Mirroring other settings of T cell aging [31], the senescence-linked loss of IL-2 expression may critically undermine activation and antiviral functions in both the T and B cell compartments [32-34]. Furthermore, CD4^+^ T cell senescence associates with constrained CD40 ligand expression and subsequent erosion of B cell helper capacity, establishing a mechanistic basis for humoral immune impairment in this context.

Nonetheless, conclusions from our single-center analysis are limited by the relatively small sample size, and therefore the CD4^+^ Tsen threshold (>19.78%) warrants additional validation in larger cohorts. We also did not evaluate senescence or function of antigen presenting and B cells which regulate vaccine responses. Finally, further investigations are expected to further delineate pathways driving T-B cell dysregulation and its implication in SARS-CoV-2 infection.

### Conclusions

In summary, our study delineates CD4^+^ T cell senescence as a reliable immune signature predicting COVID-19 vulnerability in aged patients. Future studies in larger high-risk cohorts may further define prognostic indicators and inform the design of next-generation vaccines tailored to this uniquely vulnerable population’s distinct immunological landscape.

## Supplementary Materials


