# Supplementary material for: Elevated CD4+ T Cell Senescence Associates with Impaired Immune Responsiveness in Severe COVID-19
Source: Aging Dis. 2024 Feb 14;16(1):498–511. doi: 10.14336/AD.2024.0214-2 (PMC11745426; doi:10.14336/AD.2024.0214-2)
Supplement: Supplementary file 1 [file AD-16-1-498-s.pdf]

SUPPLEMENTARY DATA

Elevated CD4^{+} T Cell Senescence Associates with Impaired Immune Responsiveness in Severe COVID-19

Jie Zhang, Chun Chang, Zhaoyuan Liang, Tingting Hu, Zhongnan Yin, Ying Liang, Ting Zhang, Yanling Ding, Xianlong Li, Xiaoyan Gai, Xiaoxue Yang, Xin Li, Xixuan Dong, Jiaqi Ren, Yafei Rao, Jun Wang, Jianling Yang, Lixiang Xue, Yongchang Sun

© 2024. Zhang J et al. Published online at http://www.aginganddisease.org/EN/10.14336/AD.2024.0214-2

Gating strategy of S1 Panel

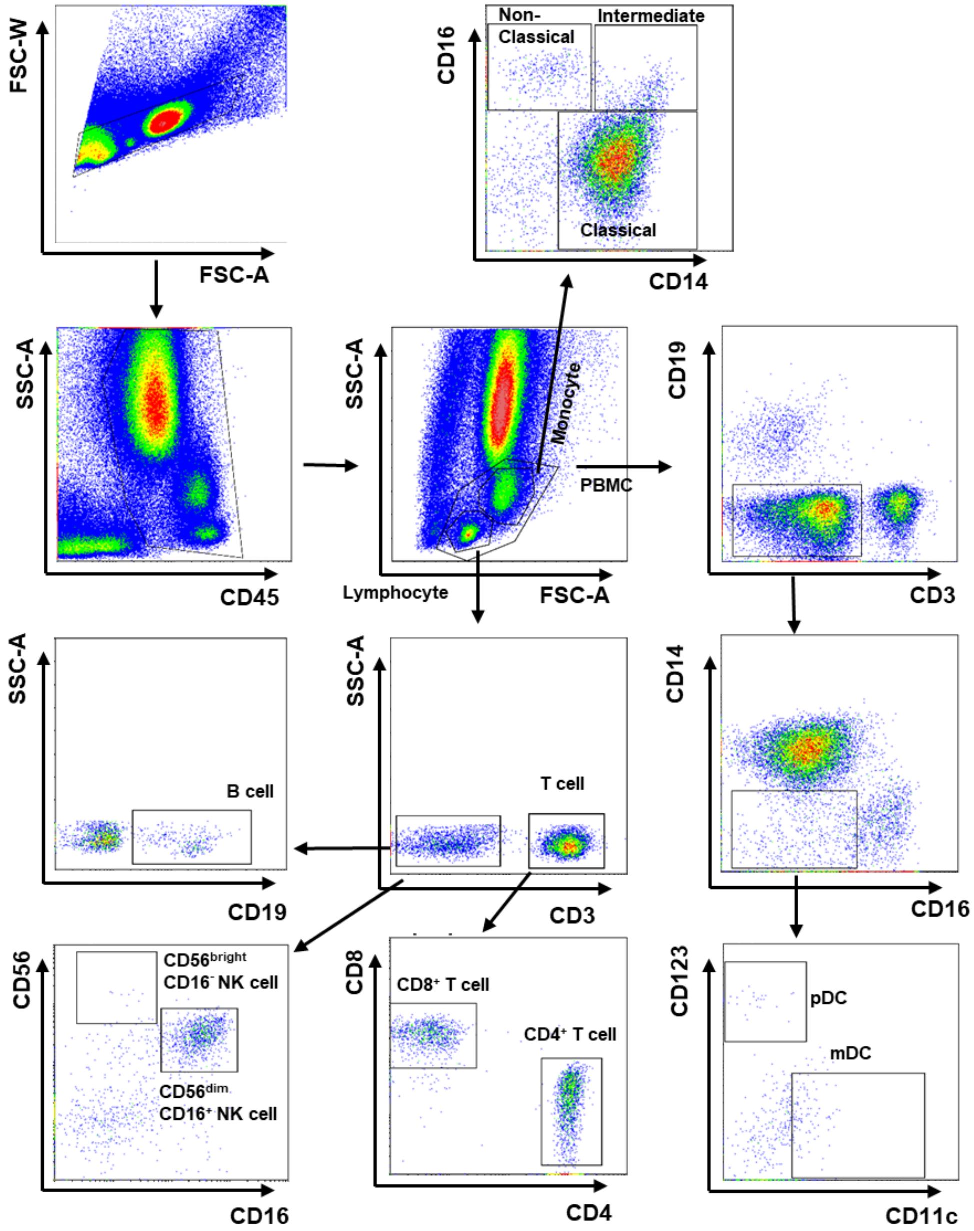

© 2024. Zhang J et al. Published online at http://www.aginganddisease.org/EN/10.14336/AD.2024.0214-2

SUPPLEMENTARY DATA

Gating strategy of S2 Panel

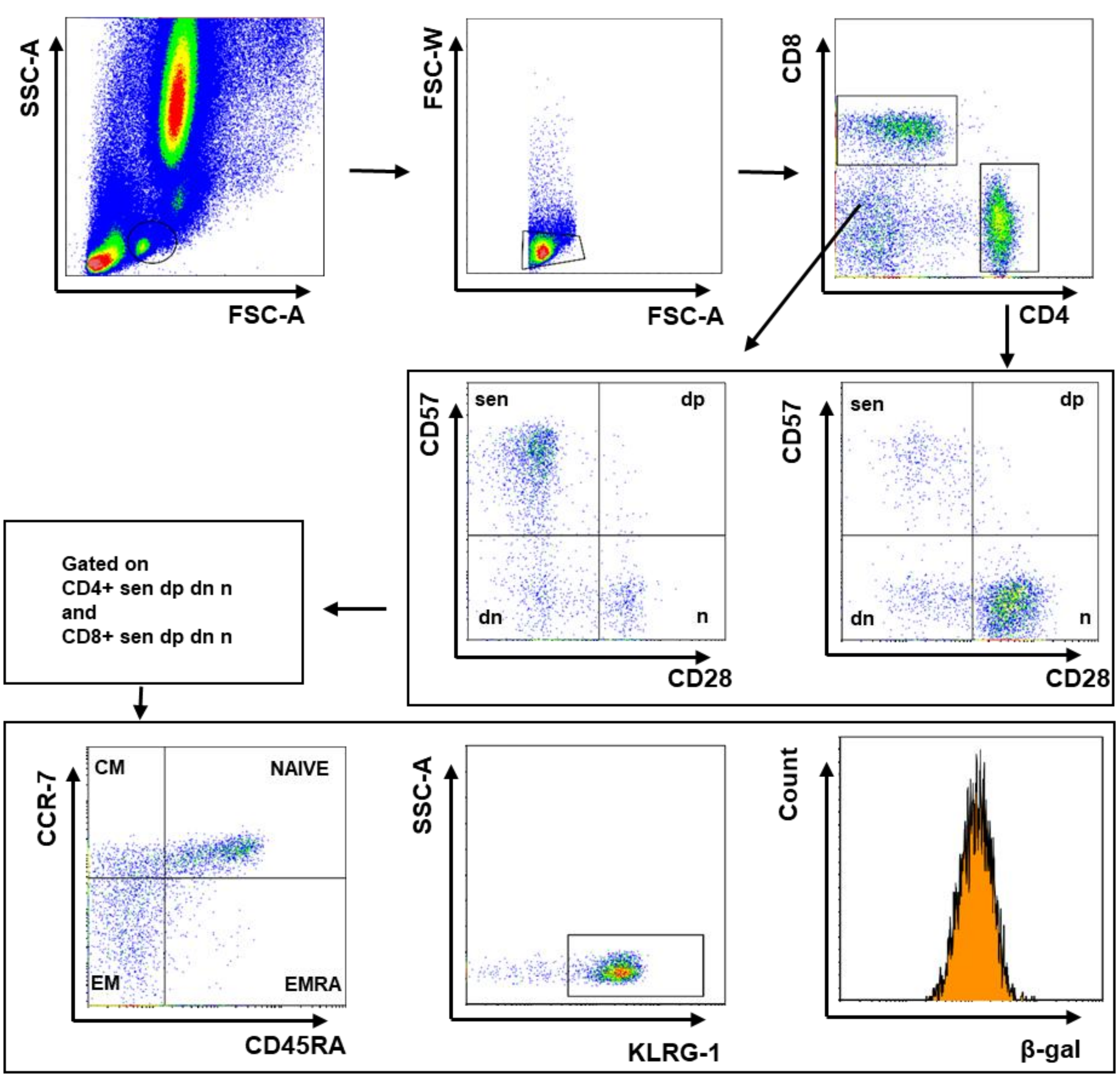

© 2024. Zhang J et al. Published online at http://www.aginganddisease.org/EN/10.14336/AD.2024.0214-2

SUPPLEMENTARY DATA

Gating strategy of S3 Panel

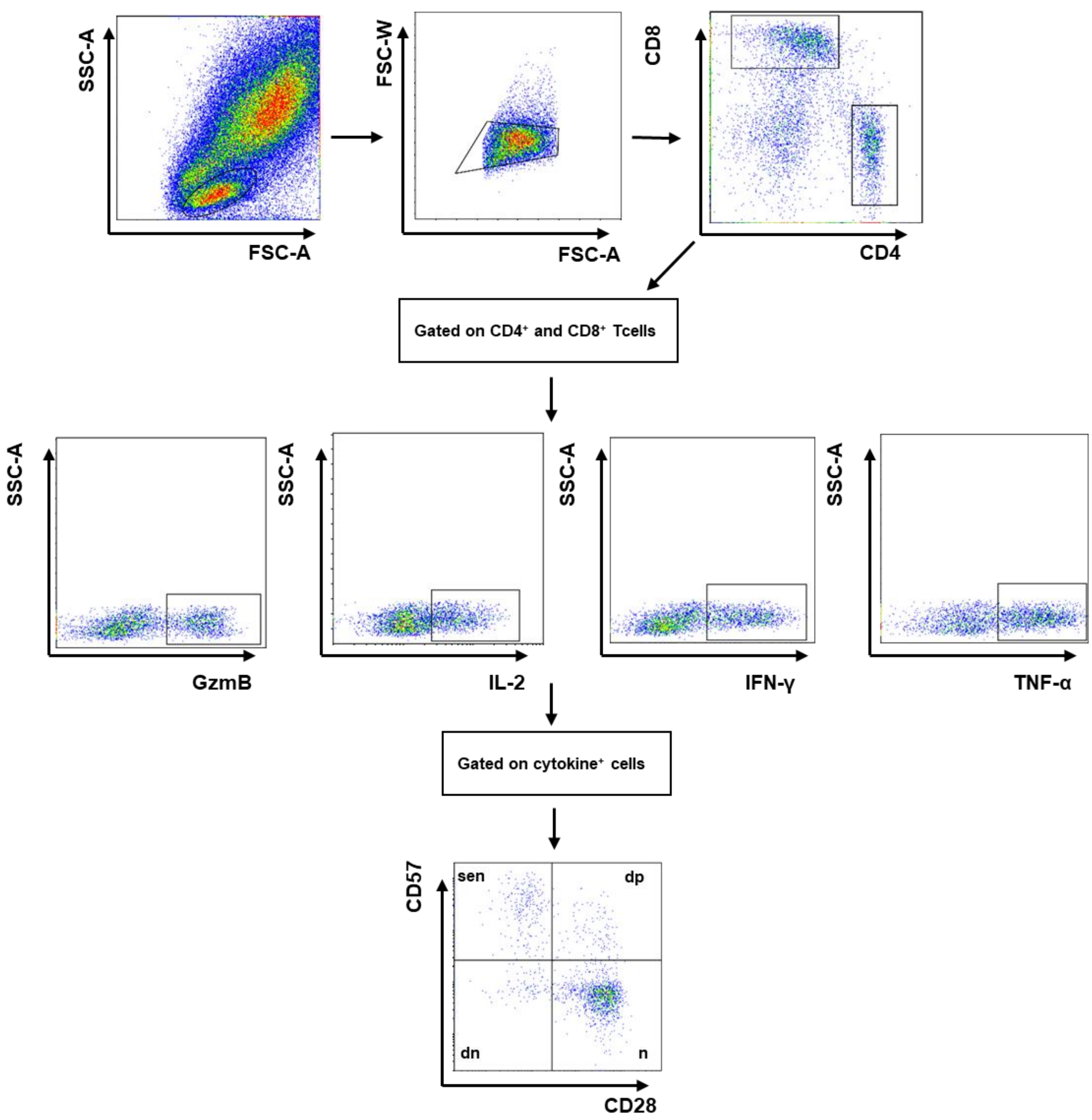

Supplementary Figure 1. The gating strategy of S1, S2 and S3 Panel.

© 2024. Zhang J et al. Published online at http://www.aginganddisease.org/EN/10.14336/AD.2024.0214-2

SUPPLEMENTARY DATA

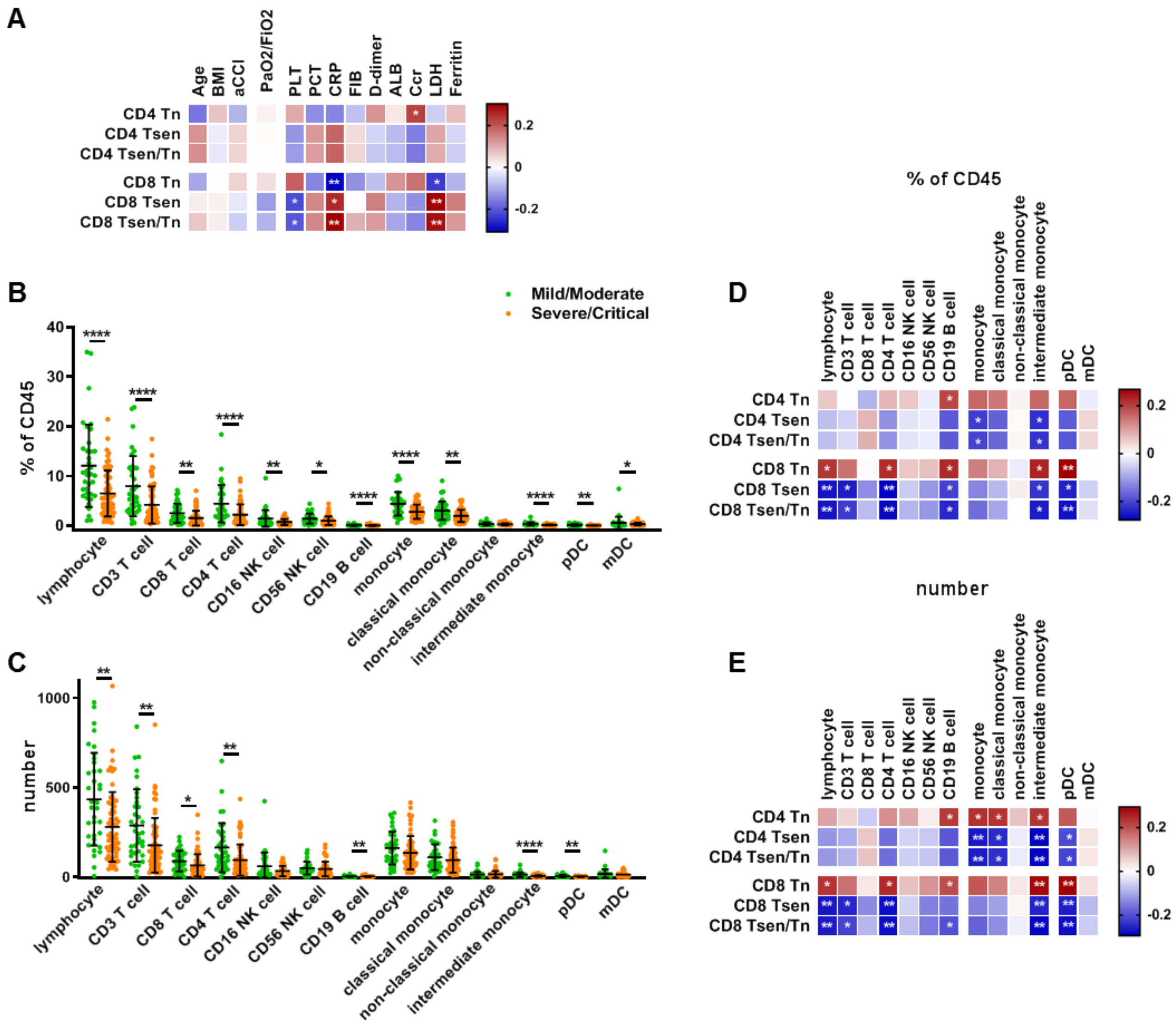

Supplementary Figure 2. (A) Correlations between the 3 subsets (Tn, Tsen, Tsen/Tn) of T cells and clinical phenotypes (n=100). (B) The percentage of different immune cell subtypes in $CD45^+$ white blood cells of mild/moderate (n=36) or sever/critical (n=64) patients. (C) The number of different immune cell subtypes in mild/moderate (n=36) or mild/moderate (n=64) patients. (D) Correlations between the 3 subsets (Tn, Tsen, Tsen/Tn) of T cells and the percentage of different immune cell subtypes in $CD45^+$ white blood cells (n=100)**.** (E) Correlations between the 3 subsets (Tn, Tsen, Tsen/Tn) of T cells and the number of different immune cell subtypes (n=100)

*, p< 0.05; **, p <0.01; ***, p <0.001; ****, p <0.0001.

© 2024. Zhang J et al. Published online at http://www.aginganddisease.org/EN/10.14336/AD.2024.0214-2

SUPPLEMENTARY DATA

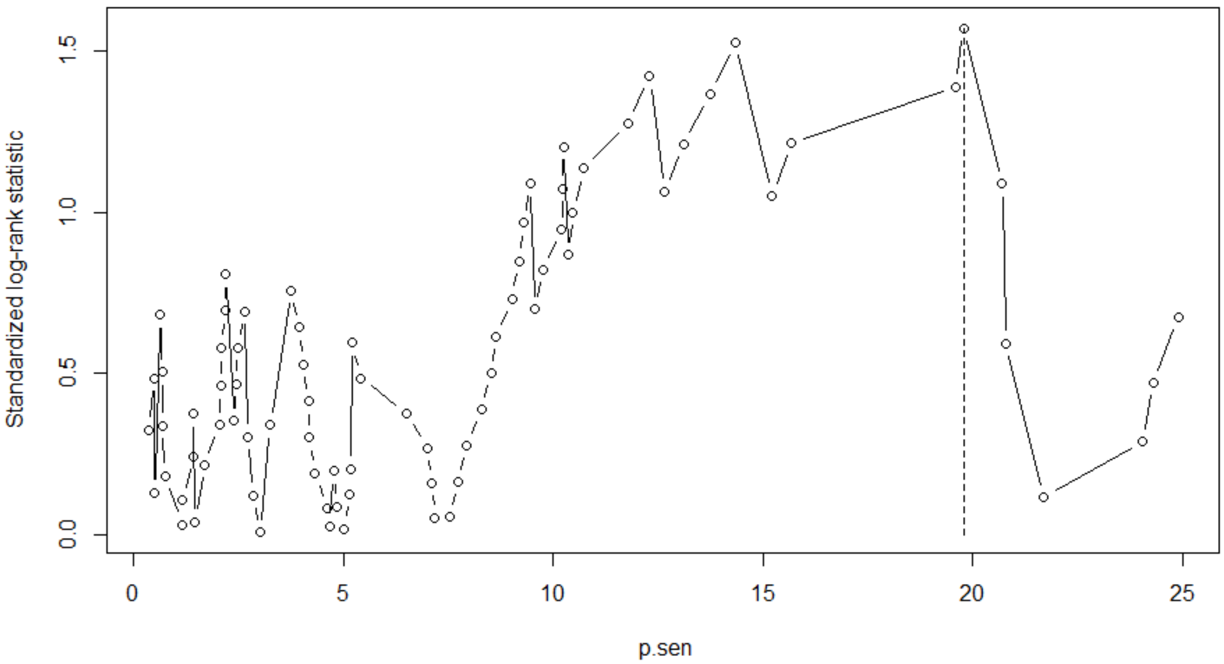

Supplementary Figure 3. Optimization of CD4 Tsen cut-off according to maximization of log-likelihood ratio method. Variation of death rate according to circulating senescent lymphocytes (% $CD28^-CD57^+$ among $CD4^+$ T-cells)

© 2024. Zhang J et al. Published online at http://www.aginganddisease.org/EN/10.14336/AD.2024.0214-2

SUPPLEMENTARY DATA

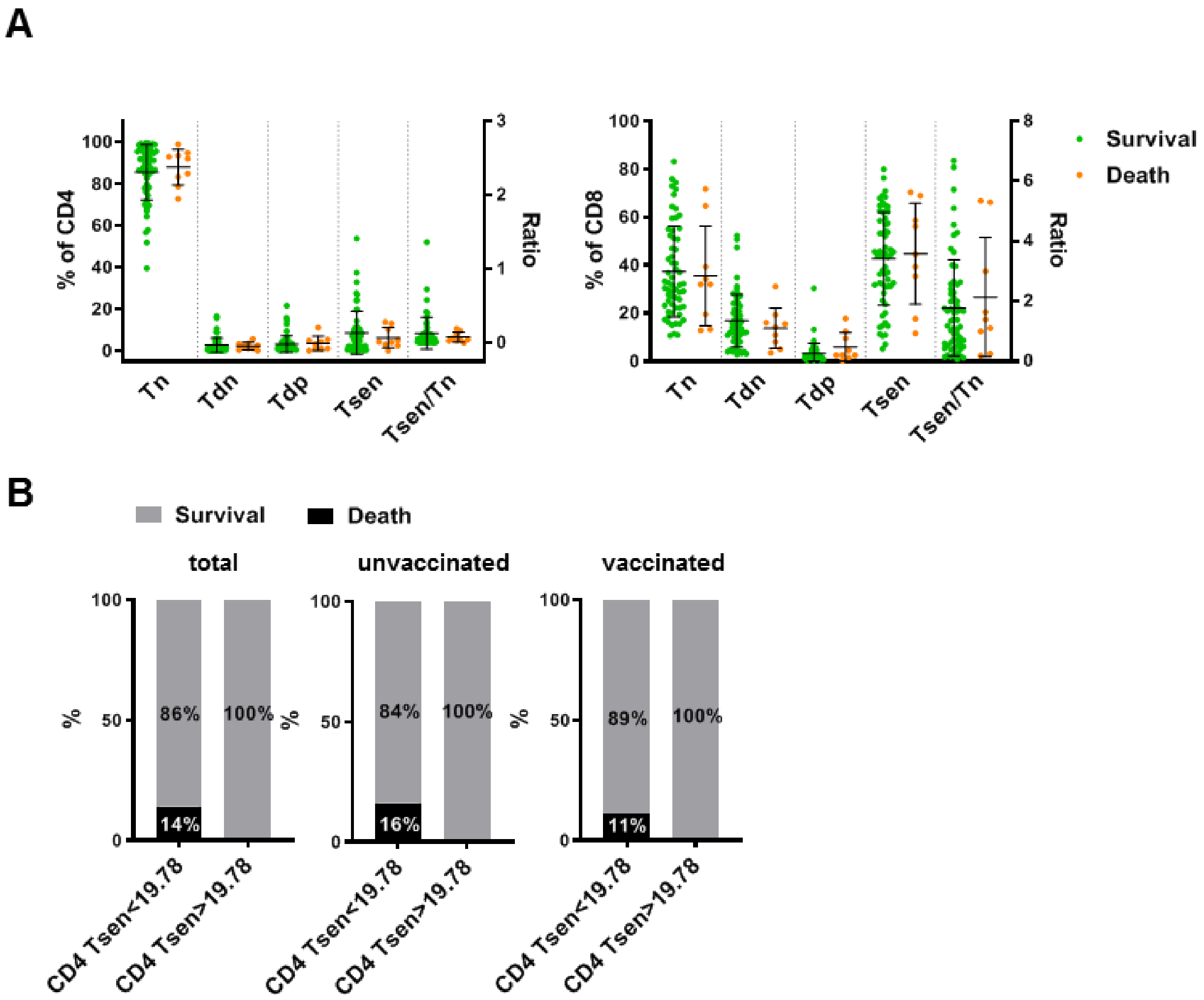

Supplementary Figure 4. CD4$^+$ Tsens was not related to long-term outcomes of COVID-19. (A) The percentage of CD4$^+$ and CD8$^+$ T cell subsets: CD28$^+$CD57$^-$ (Tn), CD28$^-$CD57$^-$(Tdn), CD28$^+$CD57$^+$ (Tdp), and CD28$^-$CD57$^+$ (Tsen) and Tsen/Tn ratio in the survival (n=61) compared to the death (n=9). Groups were compared using Mann–Whitney U-test. Bars show mean with SD. (B) Long-term (One year) survival rates in patients with COVID-19 stratified by levels of CD4 Tsen (the entire cohort: CD4 Tsen≤19.78%, n=63 versus CD4 Tsen>19.78%, n=7; unvaccinated group: CD4 Tsen≤19.78%, n=45 versus CD4 Tsen>19.78%, n=5; vaccinated group: CD4 Tsen≤19.78%, n=18 versus CD4 Tsen>19.78%, n=2). P values for difference between survival rates were calculated using Fisher exact test.

© 2024. Zhang J et al. Published online at http://www.aginganddisease.org/EN/10.14336/AD.2024.0214-2

SUPPLEMENTARY DATA

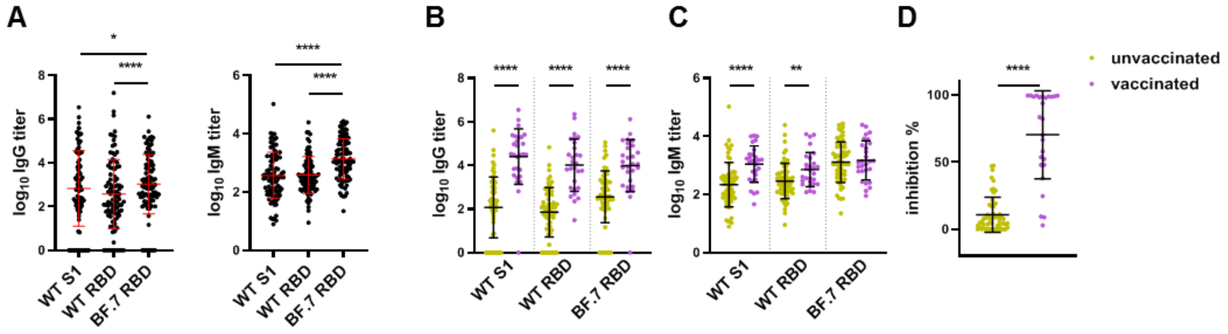

Supplementary Figure 5. The spike specific antibody titers and the inhibition rate of neutralization antibody in COVID-19 patients. (A) The titer of IgG or IgM in plasma against the protein of WT S1, WT RBD and BF.7 RBD (n=97)

(B-C) The titer of IgG or IgM against the protein of WT S1, WT RBD and BF.7 RBD in unvaccinated (n=55) or vaccinated patients (n=26). (D) The inhibition rate of neutralization antibody in unvaccinated (n=55) or vaccinated patients (n=26)

*, p< 0.05; **, p <0.01; ***, p <0.001 ; ****, p<0.0001.

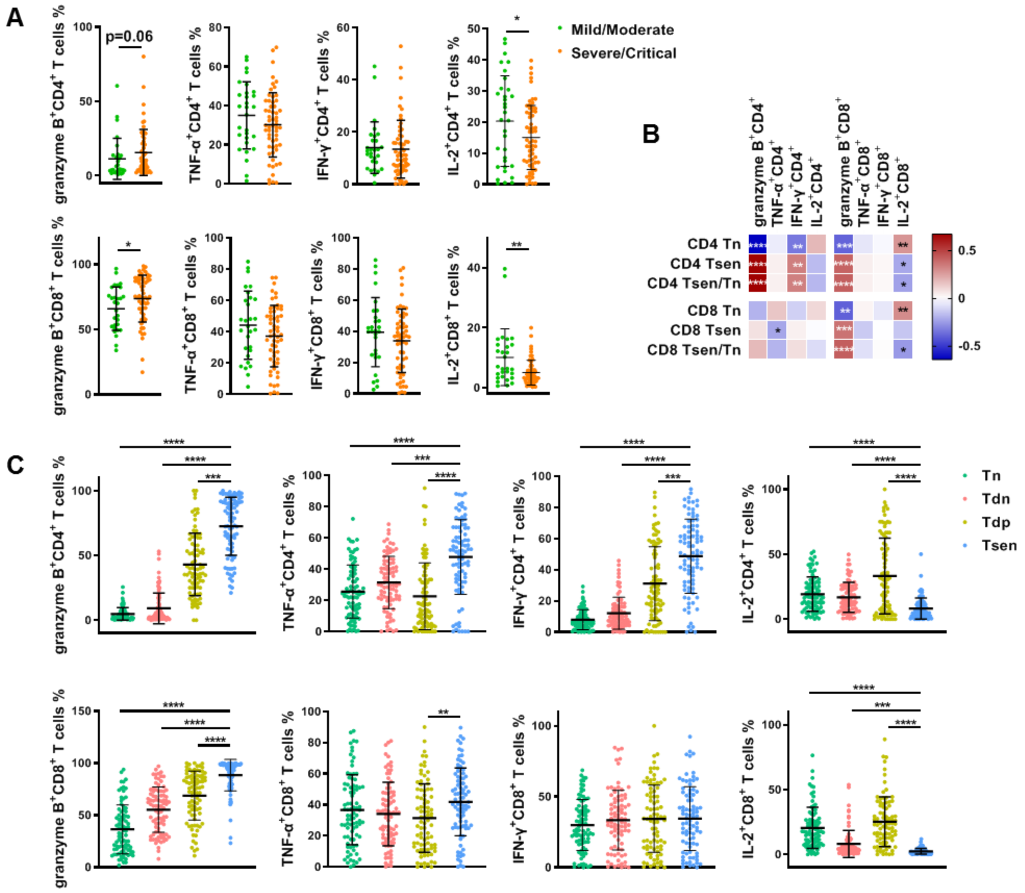

Supplementary Figure 6. The percentage of T cells that release cytokines. (A) The percentage of different cytokines (granzyme B, TNF-α, IFN-γ, IL-2) released $CD4^+$ or $CD8^+$ T cells in mild/moderate (n=29) or sever/critical (n=58) patients **.** (B) Correlations between the percentage of 3 subsets of T cells (Tn, Tsen, Tsen/Tn) and the percentage of cytokine released T cells (n=87). (C) The percentage of different cytokines (granzyme B, TNF-α, IFN-γ, IL-2) released subsets (Tn, Tdn, Tdp, Tsen) of $CD4^+$ and $CD8^+$ T cells (n=87). *, p< 0.05; **, p <0.01; ***, p <0.001 ; ****, p<0.0001.

© 2024. Zhang J et al. Published online at http://www.aginganddisease.org/EN/10.14336/AD.2024.0214-2

SUPPLEMENTARY DATA

Supplementary Table 1. Reagents used in this research.

Reagent in Different Panels	Com	Catalog	Clone
**Panel S1**			
PE anti-human CD4	Biolegend	300508	RPA-T4
PerCP-cy5.5 anti-human CD8	Biolegend	344710	SK1
PE-Cy7 anti-human CD56	Biolegend	318318	HCD56
Brilliant Violet 605 anti-human CD16	Biolegend	360727	B73.1
PE-CF594 anti-human CD14	Biolegend	325634	HCD14
APC-Cy7 anti-human CD3	Biolegend	300426	UCHT1
APC anti-human CD123	Biolegend	306012	6H6
Brilliant Violet 421 anti-human CD19	Biolegend	302233	HIB19
Brilliant Violet 510 anti-human CD11c	Biolegend	371513	S-HCL-3
Brilliant Violet 650 anti-human CD45	Biolegend	304043	HI30
**Panel S2**			
APC-Cy7 anti-human CD4	Biolegend	357416	A161A1
PerCP-cy5.5 anti-human CD8	Biolegend	344710	SK1
PE-Cy7 anti-human CD28	Biolegend	302926	CD28.2
Brilliant Violet 421 anti-human CD57	Biolegend	359608	HNK-1
Brilliant Violet 650 anti-human CD45RA	Biolegend	304135	HI100
PE-CF594 anti-human CCR7	Biolegend	353236	G043H7
APC anti-human KLRG-1	Biolegend	367715	SA231A2
Cellular Senescence Detection Kit-SPiDER-β Gal		SG03	
**Panel S3**			
APC-Cy7 anti-human CD4	Biolegend	357416	A161A1
PerCP-cy5.5 anti-human CD8	Biolegend	344710	SK1
PE-Cy7 anti-human CD28	Biolegend	302926	CD28.2
Brilliant Violet 421 anti-human CD57	Biolegend	359608	HNK-1
FITC anti-human IFN-γ	Biolegend	359607	HNK-1
PE-CF594 anti-human TNF-α	Biolegend	502946	Mab11
APC anti-human GranzymeB	Biolegend	372204	QA16A02
Brilliant Violet 605 anti-human IL-2	Biolegend	500331	MQ1-17H12
**Panel S4**			
APC-Cy7 anti-human CD4	Biolegend	357416	A161A1
PerCP-cy5.5 anti-human CD8	Biolegend	344710	SK1
PE-Cy7 anti-human CD28	Biolegend	302926	CD28.2
Brilliant Violet 421 anti-human CD57	Biolegend	359608	HNK-1
APC anti-human CD27	Biolegend	302809	O323
**Panel S5**			

© 2024. Zhang J et al. Published online at http://www.aginganddisease.org/EN/10.14336/AD.2024.0214-2

Brilliant Violet 510 anti-human CD4	Biolegend	357419	A161A1
Brilliant Violet 650 anti-human CD8	Biolegend	344729	SK1
PE-Cy7 anti-human CD28	Biolegend	302926	CD28.2
Brilliant Violet 421 anti-human CD57	Biolegend	359608	HNK-1
p21 Alexa Fluor® 488	Cell Signaling	5487	12D1
p16 PE	Cell Signaling	82548	D7C1M
**Panel S6**			
APC-Cy7 anti-human CD4	Biolegend	357416	A161A1
PerCP-cy5.5 anti-human CD8	Biolegend	344710	SK1
PE-Cy7 anti-human CD28	Biolegend	302926	CD28.2
Brilliant Violet 421 anti-human CD57	Biolegend	359608	HNK-1
APC-Cy7 anti-human CD3	Biolegend	300426	UCHT1
APC anti-human CD40L	Biolegend	310809	24-31
**Panel S7**			
CFSE	Biolegend	423801	
APC-Cy7 anti-human CD19	Biolegend	302233	HIB19
PE anti-human CD38	Biolegend	303505	HIT2
PE-Cy7 anti-human IgD	Biolegend	348209	IA6-2

Supplementary Table 2. Demographics, Characteristics, and Clinical Features of Patients With Coronavirus Disease 2019[a].

Characteristics	All cases（n=100）	CD4 Tsen low (n=84)	CD4 Tsen high (n=16)	*P*-value[b]
Age, y(n)	80.10±9.89	79.98±9.89	80.69±10.22	0.797
Sex, male	64（64%）	53（63.1%）	11（68.8%）	0.666
BMI, kg/m^2	23.81±3.91（96）	23.84±3.97	23.69±3.74	0.895
<18.5	7（7.3%）	5（6.2%）	2（13.3%）	0.578
18.5-23.9	40（41.7%）	35（43.2%）	5（33.3%）	
24.0-27.9	37（38.5%）	30（37.0%）	7（46.7%）	
≥28.0	12（12.5%）	11（13.6%）	1（6.7%）	
Smoking History，yes（n）	35（35.0%）	27（32.1%）	8（50.0%）	0.170
Any comorbidity				
Diabetes	25（25%）	22（26.2%）	3（18.8%）	0.753
Hypertension	52（52.0%）	40（47.6%）	12（75.0%）	0.045
Cardiovascular diseases	24（24.0%）	23（27.4%）	1（6.3%）	0.135
COPD	11（11.0%）	8（9.5%）	3（18.8%）	0.519
Asthma	4（4.0%）	3（3.6%）	1（6.3%）	0.508
aCCI	4.92±1.23	4.69±1.25	4.85±1.33	0.431
Signs and symptoms				
Fever	82（82.0%）	68（81.0%）	14（87.5%）	0.787
Cough	85（85.0%）	70（83.3%）	15（93.8%）	0.492
Sputum Production	80（80.0%）	66（78.6%）	14（87.5%）	0.633

© 2024. Zhang J et al. Published online at http://www.aginganddisease.org/EN/10.14336/AD.2024.0214-2

Dyspnea	60（60.0%）	52（61.9%）	8（50.0%）	0.373
Medication				
Glucocorticoids	84（84.0%）	71（81.3%）	84（84.0%）	0.743

BMI, body mass index; aCCI, age-adjusted Charlson Comorbidity Index; COPD, chronic obstructive pulmonary disease.
a. Continuous variables were presented as mean ± SD (n); categorical variables are shown as n (%). Medication and respiratory support information was recorded during entire hospital stay; other information was recorded at admission.
b. P-values were from t-test for continuous data and from χ2 test for categorical data.

Supplementary Table 3. Laboratory Characteristics on Admission for Severely and Critically Ill Patients With Coronavirus Disease 2019[a].

Characteristics	All cases（n=100）	CD4 Tsen low (n=84)	CD4 Tsen high (n=16)	*P*-value[b]
Blood routine				
White blood cell count, 10^9/L	7.56±2.9	7.42±3.05	7.64±2.77	0.797
<3.5	2（2.0%）	2（2.4%）	0（0.0%）	0.429
3.5~9.5	75（75.0%）	61（72.6%）	14（87.5%）	
>9.5	23（23.0%）	21（25.0%）	2（12.5%）	
Neutrophil count, 10^9/L	6.39±2.77	6.42±2.84	6.20±2.46	0.785
Lymphocyte count, 10^9/L	0.78±0.48	0.78±0.49	0.76±0.38	0.686
Platelet count, 10^9/L	214.62±81.04	216.29±84.38	201.75±69.30	0.492
Hemoglobin, g/L	121±28.41	121.96±32.37	114.69±17.60	0.056
Inflammatory markers				
Procalcitonin, ng/mL	0.38±1.10	0.29±0.73	0.99±2.30	0.710
<0.1	53（54.1%）	36（48.09%）	7（50.0%）	0.883
01~0.3	30（30.6%）	26（34.7%）	4（28.6%）	
>0.3	15（15.3%）	13（17.3%）	3（21.4%）	
C-reactive protein, mg/L	24.76±40.66	25.14±37.66	44.04±63.40	0.037
≤8	41（42.7%）	36（44.4%）	45（55.6%）	0.424
>8	55（57.3%）	5（33.3%）	10（66.7%）	
Coagulation function				
D-dimer, ug/mL	2.77±5.00	2.86±4.77	1.08±1.17	0.387
≤age/100	47（49.0%）	38（47.5%）	42（52.5%）	0.523
> age/100	49（51.0%）	9（56.3%）	7（43.8%）	
Serum biochemical indicators				
Serum albumin level, g/L	31.89±4.87	31.89±4.05	31.19±4.32	0.531
Creatinine, µmol/L	98.34±92.85	96.75±71.64	99.23±103.42	0.413
Serum urea nitrogen, mmol/L	9.96±8.53	10.29±9.20	8.48±4.52	0.472
Total bilirubin, µmol/L	12.02±5.66	12.52±6.28	9.67±2.96	0.227
Alanine Aminotransferase,U/L	37.49±38.22	38.50±38.75	31.85±19.36	0.525
Aspartate Aminotransferase, U/L	42.47±35.11	42.86±27.99	41.31±19.70	0.821
Creatine kinase, U/L	109.06±182.15	111.80±192.01	94.07±117.18	0.151
Creatine kinase-MB, U/L	15.56±31.62	17.11±34.32	7.50±3.03	0.024

a. Continuous variables were presented as median (interquartile range); categorical variables are shown as n (%).
b. P-values were from t-test for normally distributed continuous data and from Mann-Whitney U test for abnormally distributed continuous data.P-values were from χ2 test for categorical data.

© 2024. Zhang J et al. Published online at http://www.aginganddisease.org/EN/10.14336/AD.2024.0214-2

SUPPLEMENTARY DATA

Supplementary Table 4. Plasma soluble factors in COVID-19 patients.

Cytokines	Mild/Moderate (n=19)	Severe/Critical (n=34)	*P*-value[b-0]
IL-2	63.46±15.86	58.34±9.993	0.3852
IL4	39.51±30.01	12.17±4.094	0.2385
Il-10	5.809±2.370	12.39±2.627	0.1013
IL-6	22.02±9.565	71.54±23.36	0.1300
Il-17a	5.221±1.582	3.519±0.8046	0.2913
TNF-α	14.86±7.479	14.28±3.242	0.9345
sFas	544.0±70.80	701.1±63.76	0.1240
sFasL	18.27±3.782	13.22±1.946	0.1940
IFN-γ	123.9±48.29	180.7±49.86	0.4578
GranzymeA	171.2±95.14	102.5±19.70	0.3659
GranzymeB	467.8±133.9	473.7±101.8	0.9723
Perforin	1050±106.2	914.5±72.63	0.2859
Granulysin	2399±143.8	2423±195.5	0.9341
IL8	11.21±4.082	16.36±5.718	0.5351
IP10	522.2±182.0	1228±255.5	0.0612
Eotaxin	79.40±10.17	75.35±6.513	0.7271
TARC	40.55±7.107	38.25±6.708	0.8266
MCP1	164.4±37.54	278.2±68.7	0.2434
RANTES	953.0±99.30	931.9±119.4	0.9056
MIP1α	7.077±3.271	9.218±7.993	0.8466
MIG	492.9±111.6	758.0±141.0	0.2054
ENA78	19.69±4.668	23.79±5.070	0.5937
MIP3α	10.84±3.767	10.23±2.242	0.8816
GROα	23.08±6.923	17.57±3.826	0.4516
ITAC	30.68±5.317	61.00±14.72	0.1389
MIP1β	2.773±0.5034	4.034±1.288	0.4791
TSLP	7.891±5.190	9.460±4.710	0.8331
IL1α	37.59±13.15	26.94±8.934	0.4941
IL1β	11.53±3.404	12.43±2.684	0.8386
GM-CSF	3.230±2.045	6.398±2.057	0.3198
IFNα2	5.942±1.260	4.956±0.9000	0.5221
IL23	53.35±13.81	46.99±6.493	0.6383
IL12p40	85.19±18.50	75.34±17.31	0.7169
IL12p70	2.748±1.025	4.177±0.7668	0.2697
IL15	152.8±39.19	115.3±22.73	0.3779
IL18	89.88±32.42	137.735.91	0.3796
IL11	108.9±33.62	140.5±28.84	0.4965
IL27	73.97±16.57	66.37±18.42	0.7843
IL33	63.12±27.80	43.12±13.72	0.4729
IL5	3.911±1.476	7.316±1.396	0.1231
IL13	7.809±4.382	16.50±4.347	0.1993
IL9	23.78±15.36	11.18±1.710	0.2836
IL17F	5.537±4.154	3.267±0.609	0.4798
IL22	6.358±2.188	4.644±0.8429	0.3904

a. Different cytokine concentrations (pg/mL) of mild/moderate or severe/critical patients in plasma were presented as mean ± SEM
b. P-values were from t-test for normally distributed continuous data and from Mann-Whitney U test for abnormally distributed continuous data.

© 2024. Zhang J et al. Published online at http://www.aginganddisease.org/EN/10.14336/AD.2024.0214-2
